# Biodegradation potential of used motor oil by mixed bacterial community: optimization, emulsification activity, bioelectrochemical and metagenomics analyses using single chamber microbial fuel cell

**DOI:** 10.1186/s12934-025-02889-5

**Published:** 2025-12-28

**Authors:** Ebtehag A. E. Sakr, Nahla M. Mansour, Hanaa M. Sabaa, K. M. El-khatib, Dena Z. Khater

**Affiliations:** 1 Botany Department, Faculty of Women for Arts, Science and Education, Ain Shams University, Cairo, Egypt; 2 Gut Microbiome and Immunology Group, Chemistry of Natural and Microbial Products Department, Institute of Pharmaceutical and Drug Industries Research, National Research Centre, Dokki, Cairo Egypt; 3 Processes Development Department, Egyptian Petroleum Research Institute, Nasr City, Cairo Egypt; 4 Chemical Engineering Department, Engineering Research and Renewable Energy Institute, National Research Centre, Dokki, Egypt

**Keywords:** Used motor oil, Optimization, SCMFC, Power density, FTIR, GC–MS, Metagenomics analyses

## Abstract

**Background:**

Used motor oil (UMO) is a dangerous environmental pollutant that needs to be treated effectively. This work introduces a novel approach for producing bioelectricity and UMO biodegradation simultaneously in a single-chamber microbial fuel cell (SCMFC) using native mixed bacterial cultures.

**Results:**

Under certain conditions (2% oil, 1% peptone, 4% inoculum, 21 days), the optimized bacterial culture degraded UMO by about 80%. Through bioelectrochemical studies, a maximum voltage of 257 mV and a power density of 36.6 mW/m² were demonstrated, showing a strong correlation between UMO removal and electricity generation. Moreover, metagenomic data showed that Firmicutes, particularly *Bacillus*, dominated the biofilm at roughly 65%. Fourier Transform Infrared (FTIR) and Gas Chromatography-Mass Spectroscopy (GC-MS) verified the breakdown of complex hydrocarbon molecules, highlighting their crucial role in UMO biodegradation and bioenergy production. The effective elimination of UMOs and simultaneous power generation, supported by metagenomic and biochemical tests, showed the microbial activity and hydrocarbon breakdown.

**Conclusions:**

The results suggest SCMFC technology as a sustainable solution for managing petroleum waste while producing renewable energy.

**Supplementary Information:**

The online version contains supplementary material available at 10.1186/s12934-025-02889-5.

## Introduction

Pollution caused by petroleum hydrocarbons (PHs) and their byproducts is a persistent worldwide issue with related disputes [1]. Used motor oil (UMO) is a complex mixture of aliphatic PHs compounds that are represented mainly by alkanes, unsubstituted and substituted C_18_ to C_47_ monocyclic, polycyclic and heterocyclic aromatic compounds. It is mainly derived from the automobile industry, including auto mechanic workshops [2]. One of the most significant environmental problems facing the ecosystem is the deliberate direct liberation of discarded UMO into soils and streams [3, 4]. As a result, numerous plans have been addressed and eradicated these hazards. Due to their high cost, time expenditure, and high energy requirements, the present conventional techniques have multiple drawbacks [5]. Thus, in major oil-producing countries, efforts toward developing new or improving biological treatments are often seen as superior solutions to other remediation approaches.

Few studies have examined the biodegradation of motor waste, despite the fact that several studies have been conducted on crude oil, petroleum industry oil sludge, and oil spills in marine environments. The most bioremediation technique is generally the use of bacteria to clean oil-contaminated locations. Unfortunately, soil is contaminated by unintentional spills or improper handling and disposal of UMO products, which occur in car repair shops and gas stations. The potential for bioremediation by UMO-degrading bacteria in soil patches surrounding car garages and gas stations is not well understood [6]. As a result, the demand for a safe and efficient UMO removal method can be addressed by employing microbial fuel cells (MFCs), which can break down wastes with the help of their anodic biofilm to produce green energy [7]. Utilizing anodic bacterial populations to break down chemical energy from waste and transform it into protons and electrons via their metabolic route is the fundamental idea behind the MFC method. Green energy is produced when the resultant electrons are subsequently moved via an external circuit into the cathodic electrode [8].

The use of PHs as a low-grade substrate and an electricity source in MFC has attracted attention in recent years. However, there is no study on the UMO bioremediation through MFC techniques. For this purpose, Permana et al. [9] executed a comparative study between single-chambered and double-chambered MFCs. They found that the single-chambered MFC can not only degrade more PHs than the dual-chamber system but also produce higher electrical energy on average than the others. In addition, Umar et al. [10] reported that xylene can be completely converted to non-toxic carbon dioxide through double-chamber benthic MFCs. These results indicate that the refractory organic matter can be decomposed by microorganisms in the MFC systems, particularly in the anodes. Moreover, Anwar Ahmad et al. [11] used MFC for petroleum wastewater (PWW) with pretreated activated sludge for the production of electricity and removal of chemical oxygen demand (COD). The MFC system resulted in the reduction of COD by 89.5% of the original value and generated electricity equivalent to 8.18 mAm^− 2^. Nicola Lovecchio et al. [12] also engineered a novel MFC that integrated the biofilm’s natural properties into the MFC design. These biofilms, composed of specialized hydrocarbon-degrading bacteria, are vital in supporting electron transfer, significantly enhancing the system’s power generation. The system showed remarkable long-term stability, which maintained consistent performance over a 5 d testing period, and sustained power output for up to 7.5 h. Varnava et al. [13] evaluated the potential of *Pseudomonas citronellolis* 620 C strain to generate electricity in a double chamber MFC, with oily wastewater as fuel. Both electrochemical and physicochemical results suggested that this strain utilized efficiently the oily wastewater and generated electricity in the MFC setup, reaching 0.05 mWm^− 2^ maximum power.

In general, the anodic electroactive microorganisms offer a variety of benefits because of their high remediation impacts, power output, and effective electron transfer from their cells into the anodic electrode via both direct and indirect pathways [14]. Because they can degrade a variety of natural substrates, tolerate harsh environmental conditions, and may release hydrolytic enzymes and biosurfactants to break down complex biomass [15]. According to a recent study by Zhuang et al. [16] revealed that exposure to high concentrations of petroleum chemicals (C_10_–C_40_) reduces the variety of soil microbes. The authors found that the higher petroleum component concentrations were associated with higher levels of diversification. Thus, to better understand the MFC principle, it is essential to investigate the dominating microbial diversity during the metabolism of UMO in MFCs utilizing high-throughput sequencing [17, 18]. To our knowledge, the electrochemical activity of our mixed bacterial community derived from an automobile mechanic workshop has never been reported through MFC techniques.

**Fig. 1:**
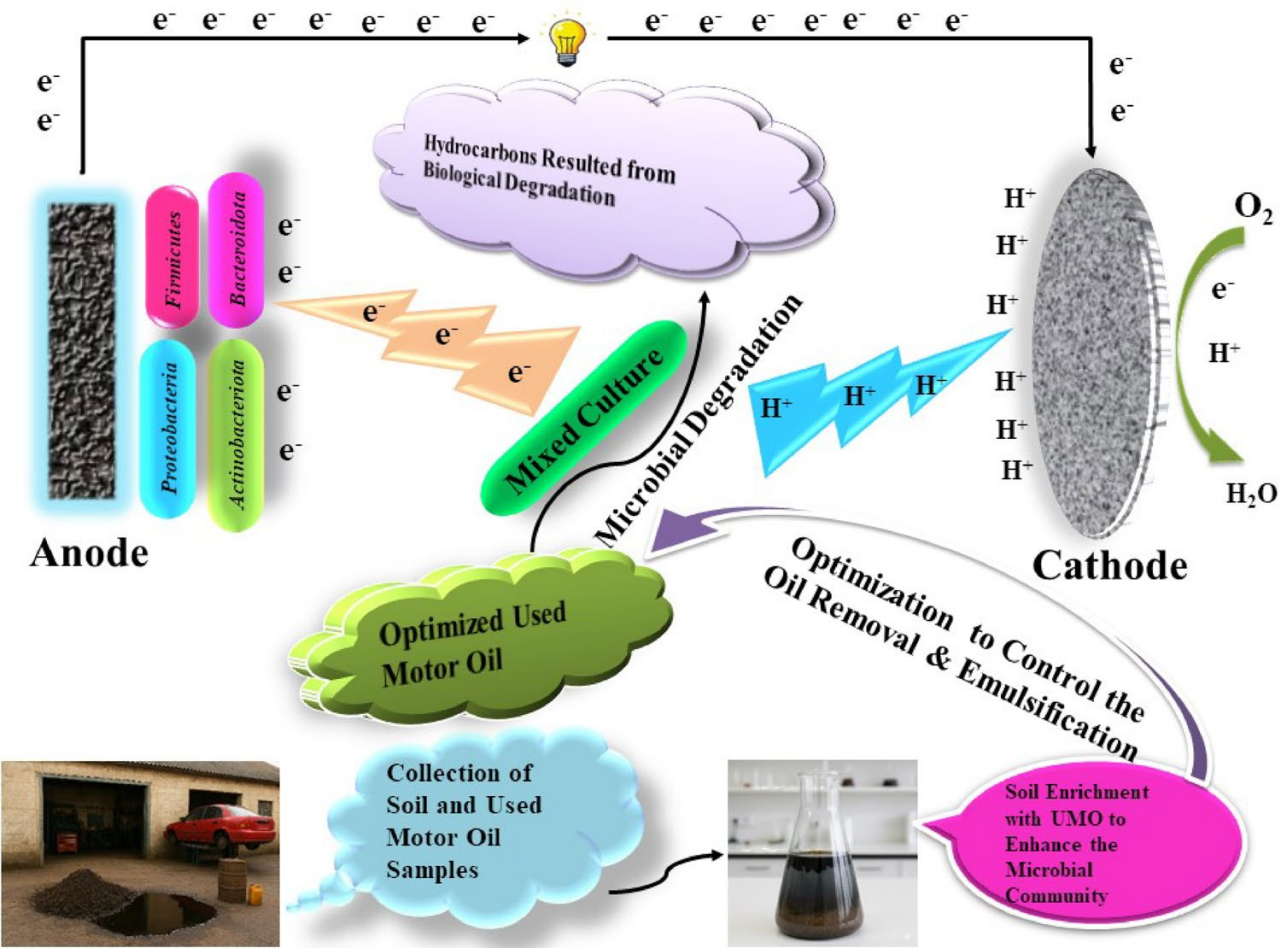
Schematic diagram for the experimental study

Herein, this work in our knowledge offered a viewpoint on the degradative activity of mixed bacterial communities, to effectively remove and convert resistant UMO as hydrophobic chemicals into green energy, utilizing MFC to lessen their negative effects. The gravimetric approach is used to assess the effects of various initial UMO, peptone, sodium acetate, inoculum size, and incubation time on biodegradation efficiency. Additionally, a single-chamber microbial fuel cell (SCMFC) operated at an optimal UMO concentration was used to evaluate the bioelectrochemical analyses for simultaneous UMO degradation and bioelectricity generation through power output, cyclic voltammetry and electrochemical impedance spectroscopy measurements. Further, Fourier transform infrared (FTIR) and Gas chromatography-mass spectroscopy (GC-MS) analyses were used to provide light on the variety of hydrocarbons that were broken down by the mixed bacterial communities. Lastly, high-throughput sequencing was used to identify the metagenomics studies of the prevalent microbial diversity. Thus, this study may be a viable for UMO bioremediation and electricity generation through MFC, simultaneously at heavily contaminated areas and industrial large scale (Fig.1).

## Results

### Enrichment of bacterial mixed culture

A variety of indigenous bacterial populations that were linked to the breakdown of petroleum hydrocarbons (PHs) were obtained and isolated from the mechanic workshop. The present work enriched these bacterial populations that break down UMO as the only source of carbon and energy. To increase the activity of the mixed bacterial community towards UMO removal, bacterial enrichment is an essential step. Throughout the degrading processes, the soil-borne microbes grew, multiplied, and expanded in number, proving their capacity to use UMO.

### Optimization of the UMO removal and bioemulsifier activity

#### Impact of initial UMO concentrations

According to the study, the maximum biodegradation efficiency was 62.73 ± 0.55% when the initial UMO concentration was 2% (v/v). This indicated that the bacterial cultures were able to metabolize and break down the oil content, reflecting effective microbial degradation activity. At 8% (v/v) UMO, an immiscible oil layer created a physical barrier that limited microbial access to the substrate, hindering biodegradation. Bacterial culture cell-free supernatants demonstrated stable UMO and vegetable oil emulsions with significant bioemulsifying activity; after 24 h, the emulsification indices for UMO and vegetable oil ranged from 22.9% to 95.2% and 12.5% to 66.3%, respectively. At 2% initial oil, the maximum emulsification activity was 62.5% for vegetable oil and 75.17% for UMO.

#### Impact of peptone and sodium acetate concentrations

The effect of peptone concentrations on UMO biodegradation in mixed bacterial cultures was evaluated in this study. At the ideal concentration of 1%, 74.83 ± 0.47% UMO removal was achieved. Increasing peptone conc. reduced biodegradation, likely due to nitrogen excess limiting biomass growth. At both greater and lower concentrations, biodegradation rates decreased. Sodium acetate did not significantly accelerate UMO decomposition. With the highest emulsification index at 1% peptone and significant emulsifying activity at 0% sodium acetate, the nitrogen supply also had an impact on the bioemulsifier’s activity.

#### Impact of inoculum size

Nutrient intake and biodegradation efficiency were highly influenced by the size of the bacterial inoculum; at a 4% (v/v) inoculum, the UMO highest rate of 78.18 ± 0.66% was recorded (Table 1). Degradation efficiency decreased with an increase to 6%, most likely because smaller inocula require longer growing durations to reach effective activity. Furthermore, when the inoculum size grew from 4% to 6%, the emulsification index (EI_24_) decreased from 94.67 ± 1.53% to 68.50 ± 1.32%, indicating less bioemulsifier synthesis at greater proportions.

**Table 1:** Optimization of the UMO removal and bioemulsifier activity percentages

Different parameters	UMO Removal (%)	EI_24_%	
		Vegetable oil	UMO
Initial concentration of used motor oil (v/v, %)			
2	62.73 ± 0.55^a^	62.50 ± 1.50 a	75.17 ± 1.26a
4	19.42 ± 0.73 ^c^	52.5 ± 0.50b	70.33 ± 0.58b
6	20.69 ± 0.52 ^b^	51.23 ± 1.07b	62.83 ± 0.95c
8	11.00 ± 0.50 ^d^	42.5 ± 0.49c	49.78 ± 0.79d
F-value	4806.37 ***	205.65***	427.14 ***
Peptone concentration (w/v, %)			
1	74.83 ± 0.47 a	65.80 ± 0.72 a	74.67 ± 2.52 a
2	59.28 ± 0.58 b	55.00 0.90 b	65.67 ± 1.15 b
3	46.17 ± 0.65 d	50.10 ± 0.17 c	63.17 ± 0.76 b
4	47.50 ± 0.98 c	46.80 ± 0.26 d	52.73 ± 0.87 c
F-value	1071.05 ***	577.81 ***	108.36 ***
Sodium acetate (w/v, %)			
0	74.35 ± 0.67 a	66.27 ± 1.10 a	74.67 ± 1.52 a
0.5	33.15 ± 0.23 b	55.37 ± 0.55 b	37.23 ± 0.83 b
1.0	23.10 ± 0.53 c	43.96 ± 0.91 c	37.30 ± 0.92 b
1.5	18.53 ± 0.68 d	33.07 ± 0.55 d	29.47 ± 0.50 c
2.0	12.32 ± 0.48 e	30.43 ± 0.75 e	22.9 ± 0.78 d
F-value	6189.11 ***	1066.57 ***	1284.03***
Inoculum size (v/v, %)			
2	70.40 ± 0.53 b	51.07 ± 1.00 b	75.27 ± 0.64 b
4	78.18 ± 0.66 a	55.67 ± 0.98 a	94.67 ± 1.53 a
6	63.47 ± 0.81 c	47.87 ± 0.32 c	68.50 ± 1.32 c
F-value	354.74 ***	66.21 ***	369.22 ***
Incubation time (d)			
7	53.18 ± 0.76 c	12.50 ± 0.30 c	44.53 ± 0.40 c
14	77.31 ± 0.77 b	60.55 ± 0.62 b	87.47 ± 0.55 b
21	79.54 ± 0.45 a	65.77 ± 0.67 a	95.17 ± 0.76 a
F-value	1397.88***	8408.16 ***	6380.42***

UMO: used motor oil

EI_24_%; Emulsification index

#### Impact of incubation time

Over time, UMO biodegradation improved dramatically, reaching a peak of 79.54 ± 0.45% after 21 d, compared to 53.18 ± 0.76% after 7 d. The Duncan test, in particular, revealed a considerable drop in UMO levels between these time intervals, supporting that 21 d is the ideal duration for biodegradation. For the mixed culture to thrive on UMO, the incubation duration is crucial, as evidenced by the highest EI_24_ at 21 d. Additionally, because lipases and biosurfactants use UMO as their only source of carbon and energy, the obvious emulsification of culture medium provides evidence that the synthesis of extracellular biosurfactants and bioemulsifiers contributes to the breakdown of UMO.

### Correlation between UMO removal and bioemulsifier activity

The regression analysis in Table 2 confirmed that the EI_24_ index and UMO removal were directly correlated. Through the formation of certain metabolites that emulsify the vegetable oil and UMO, the bacterial mixture was in charge of using UMO as a source of carbon in the BMS medium since the amount of UMO decreased as a result of an increase in cell numbers. Regression analysis was therefore carried out to determine the relevant relationships. The emulsification activity of the culture broth and UMO biodegradation showed a strong correlation, suggesting that the production of emulsifying agents by a mixed bacterial culture may improve waste oil uptake.

**Table 2:** Pearson correlation between UMO removal and bioemulsifier activity

	UMO Removal (%)	EI_24_%	
		Vegetable oil	UMO
Initial concentration of used motor oil (v/v, %)			
UMO Removal (%)	1		
EI_24_% with vegetable oil	0.92**	1	
EI_24_% with UMO	0.75**	0.93**	1
Peptone concentration (w/v, %)			
UMO Removal (%)	1		
EI_24_% with vegetable oil	0.97**	1	
EI_24_% with UMO	0.85**	0.93**	1
Sodium acetate (w/v, %)			
UMO Removal (%)	1		
EI_24_% with vegetable oil	0.91**	1	
EI_24_% with UMO	0.99**	0.89**	1
Inoculum size (v/v, %)			
UMO Removal (%)	1		
EI_24_% with vegetable oil	0.96**	1	
EI_24_% with UMO	0.96**	0.97**	1
Incubation time (d)			
UMO Removal (%)	1		
EI_24_% with vegetable oil	0.99**	1	
EI_24_% with UMO	0.99**	0.99**	1

**Correlation is significant at the 0.01 level (2-tailed)

UMO: used motor oil

EI_24_%; Emulsification index

In the initial UMO concentrations, UMO removal was significantly positively correlated with EI_24_% with vegetable oil (*p* < 0.01), UMO removal was significantly positively correlated with EI_24_% with UMO (*p* < 0.01), and EI_24_% with vegetable oil was significantly positively correlated with EI_24_% with UMO (*p* < 0.01). Moreover, the same trends of Pearson correlation were found with peptone, sodium acetate, inoculum size, and incubation time.

### The bioelectrochemical performance

#### Voltage generation

**Fig. 2:**
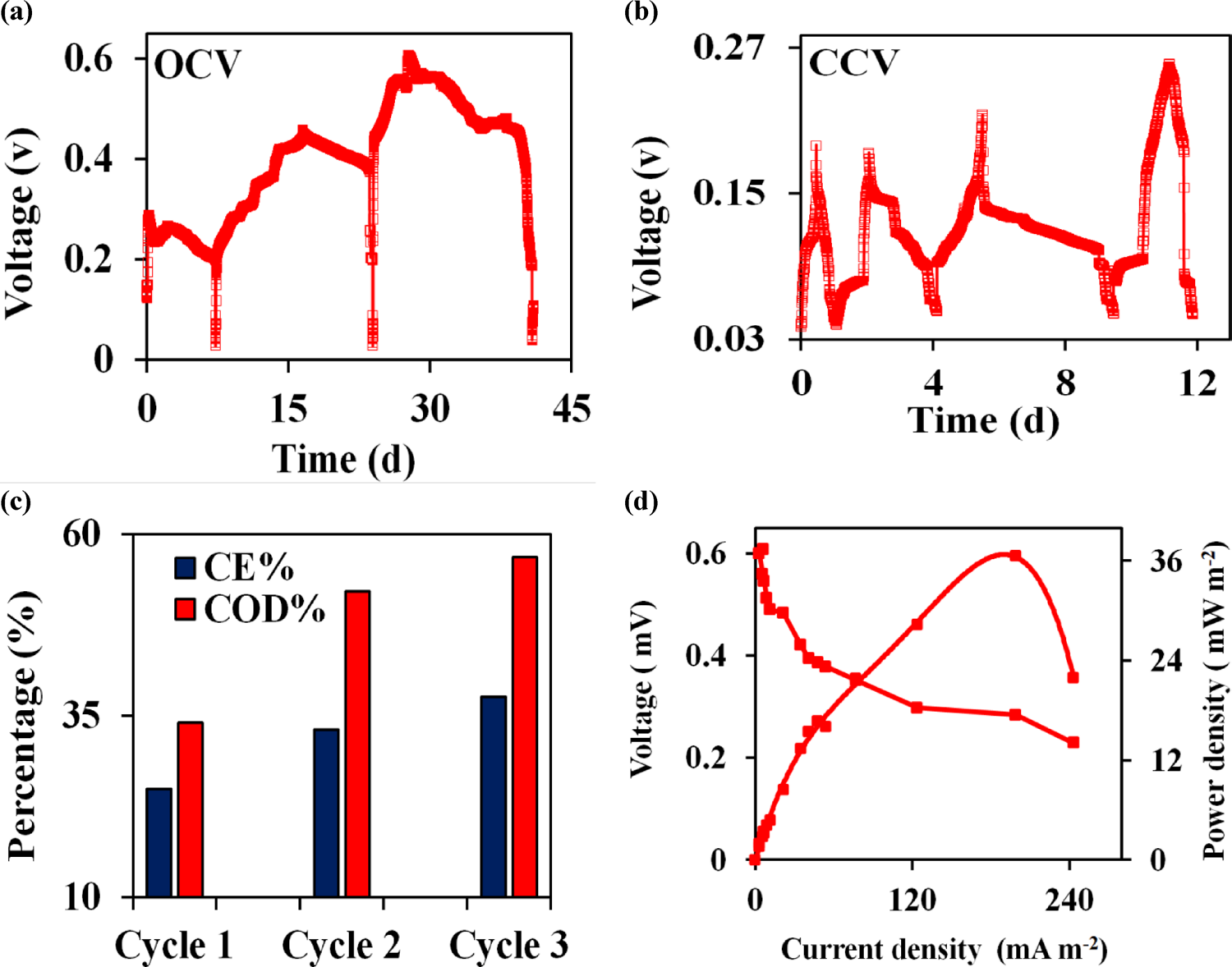
**a) **Voltage vs. time (OCV) curve, **b)** Voltage vs. time (CCV) curve at 10000 Ω; **c** ) COD removal efficiency and CE of UMO for three batch cycles; **d)** Profile of current densities and power densities of 2% raw UMO in SCMFC

The mixed bacterial cultures were used in operation and amended with UMO as an energy source at an optimum maximum tolerable concentration of 2% under batch operation. The single chamber microbial fuel cell (SCMFC) was then left for 42 d with open circuit voltage (OCV), as shown in Fig. 2a. It can be observed that the voltage development throughout the experiment vs. time was gradually increased and documented during the stable operation phase. As seen, in the first cycle, at the initial stage of biofilm progress, the maximum OCV levels were progressively reached approximately 287 ± 0.04 mV after a duration time of 6 h. Then, a stable pattern of the voltage began to decrease to reach 50 mV within 10 d due to the formation of a stable anodic biofilm. Afterward, the SCMFC was refreshed with UMO addition in the following batch cycle, and the OCV was increased to 457 ± 0.013 mV with a duration time of almost 17d. This cycle had the same manner as the first cycle and the OCV began to be reduced after approximately 24 d. Moreover, the overall maximum OCV production was 608 ± 0.03 mV after 28 d of acclimation of the anodic microbial community during the first three cycles.

During the course of the experiment, after six months, an external resistance of 10,000 Ω was applied for a total of 13 d on the SCMFC that was inoculated with a newly prepared 2% UMO medium to determine the proficiency of the SCMFC in generating electrical energy from UMO. It was reported that the close circuit voltage (CCV) was reduced to 257 ± 0.01 mV at final COD concentration of 2.25 ± 0.51 gL^− 1^ with a duration time of almost 11 d, which was the maximum measured value as illustrated in Fig. 2b. In addition, the chemical oxygen demand (COD) removal efficiency and coulombic efficiency (C_E_%) for the treatment of UMO was analyzed for all the three cycles at the end of each cycle. Figure 2c depicts a comparison of the COD removal efficiency and C_E_% of SCMFC that was inoculated with 2% UMO at an initial concentration of 41.5 g COD L^− 1^ and subjected under an external resistance of 10,000 Ω for 13 d. It was obvious that there was a proportional relationship between COD removal and C_E_%. Primarily, both the COD removal and C_E_% efficiencies of the SCMFC system were very low, but a significant increase in both parameters was found as the cycles progressed due to the formation of a stable biofilm over the electrode.

Referring to Table 3, where the SCMFC characteristics at steady states during its whole operation were presented, the high COD removal efficiency rates were 34.08, 52.19 and 56.88% which attributed to the long operation period; a consequently, the high corresponding C_E_% of 24.95, 33.12 and 37.59%.

**Table 3:** Steady state electrochemical characteristics of UMO during the SCMFC whole operational period

	CCV (mV)	COD removal efficiency (%)	C_E_ (%)	UMO removal %	EI_24_%	
					Vegetable oil	UMO
Cycle 1	176 ± 0.03	34.08	24.95	84.18 ± 0.58	57.43 ± 0.60	67.17 ± 1.04
Cycle 2	215 ± 0.05	52.19	33.12	77.43 ± 0.67	52.70 ± 0.72	62.86 ± 0.47
Cycle 3	257 ± 0.01	56.88	37.59	80.90 ± 0.78	62.83 ± 0.95	50.66 ± 1.08

Values were means of triplicate experiments ± SD

CCV; close circuit voltage

COD; chemical oxygen demand

C_E_%; coulombic efficiency

UMO; used motor oil

EI_24_%; Emulsification index

#### Polarization and power density curves

Furthermore, the polarization and power density curves were obtained for SCMFC inoculated with 2% raw UMO and subjected to a wide range of different external resistances ranging from 100 k Ω to 50 Ω in descending order to assess the performance of SCMFC towards UMO bioremediation on the basis of current generation. The relationships between current density (CD), power density (PD), and voltage (V) were estimated, normalized to the anodic active surface area and plotted in Fig. 2d to show the physiological behavior of anodic biofilm in the used SCMFC. As illustrated in Fig. 2d, there were three phases in the polarization curve of log, stationary, and decline phases. At the beginning, the acclimatization period, there was a decrease in voltage output that could be attributed to the activation loss and sluggish electron transfer rate according to the log phase of the formed anodic biofilm. During the second phase, the stationary phase, the CD rose and a linear relationship of voltage and current was demonstrated due to ohmic losses. Hence, the SCMFC stabilized and achieved maximum levels of power densities. The maximum PD reached its highest value of 36.60 mW m^− 2^ at an optimum CD of 243.24 mA m^− 2^. Following this, there was a steep drop in power density values, which could be attributed to mass transport losses resulting from the loss of UMO in the media and the decline phase of the anodic biofilm. Thus, to restore the availability, the SCMFC was refreshed with new UMO wastewater. On the anodic biofilm surface, the UMO was consumed and oxidized anaerobically by microorganisms, producing electrons and protons. Electrons and protons were turned from the anodic biofilm to the cathodic electrode, where the reduction of an oxidant, aerobically, occurred. In a system, as illustrated in the following Eqs. (1–2)

Anodic biofilm surface:

1 $${\mathrm{nCH}}_{{\mathrm{2}}} {\text{O }} + {\text{ nH}}_{{\mathrm{2}}} {\text{O }} \to {\text{ nCO}}_{{\mathrm{2}}} + {\text{ 4ne}}^{ - } + {\text{ 4nH}}^{ + } ~~~$$

Cathodic surface:

2 $$nO_{2} + {\text{ }}4ne^{ - } + {\text{ }}4nH^{ + } \to {\text{ }}2nH_{2} O~~~$$

#### Electrochemical characterization

##### Cyclic voltammetry

**Fig. 3:**
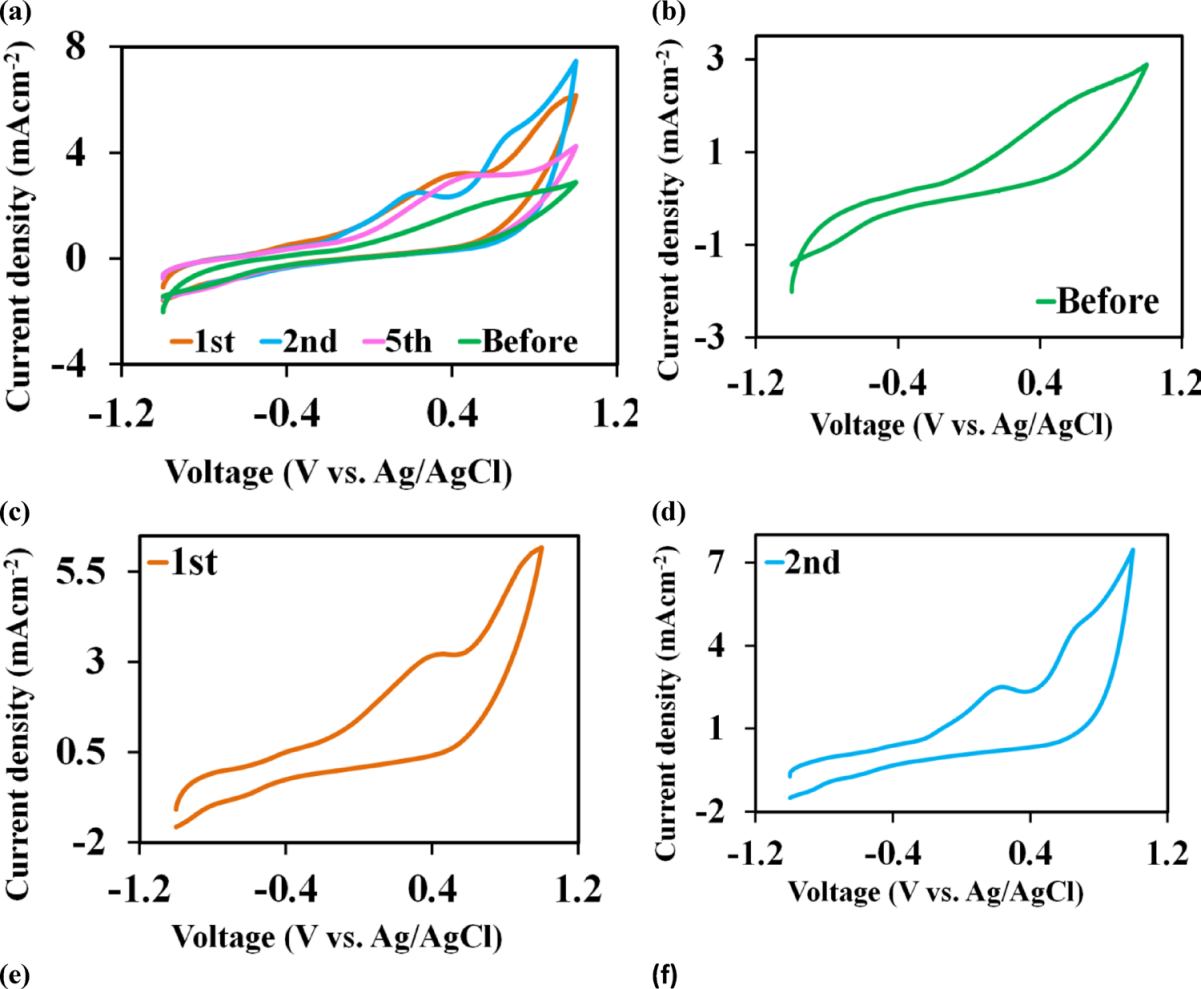

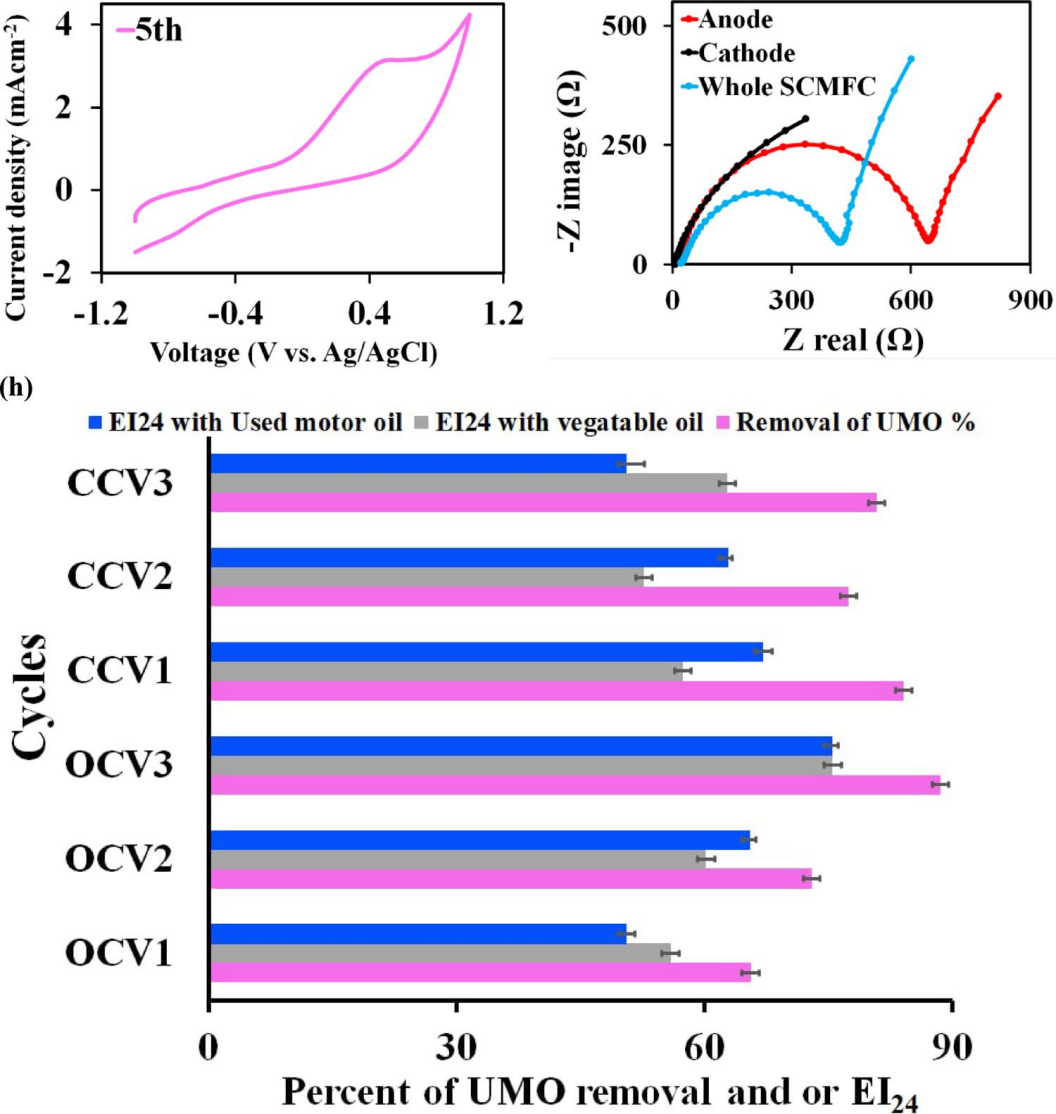
**a) **Cyclic voltammogram was recorded at a voltage sweep values from – 1.0 to 1.0 V vs. Ag/AgCl reference electrode at various time intervals ( 0 h and 1, 2, 5 days) at a scan rate of 5 mVs^−1^; Cycles are shown here as a clockwise direction **b)** CV before inoculation; **c)** first day; **d)** second day and **e)** fifth day.** f ) **The Nyquist plot for anodic, cathode and whole SCMFC inoculated with 2% raw UMO at 41.5 g COD L^−1^, using the anode or the cathode as working electrode and Ag/AgCl (3 M KCl) as reference electrode.** h) **UMO removal and EI_24_% of cell free supernatant obtained from open and closed circuit voltage cycles

Cyclic voltammetry (CV) was used to study the bioelectrocatalytic behavior of the formed biofilm towards the UMO oxidation in the SCMFC bioreactor at steady state OCV. It was recorded in situ at a voltage sweep from – 1.2 to 1.2 V vs. Ag/AgCl reference electrode at various time intervals (0 h and 1, 2, 5 d) at a scan rate of 5 mVs^− 1^. The voltammetry profiles over the batch cycle at various times throughout the operation were illustrated in Fig. 3a. Noticeably, as described in Fig. 3b, the CV displayed a smooth curve before inoculation with no redox peaks during the oxidation andreduction process that might be due to unformed immature biofilm on the anodic surface and was hence not considered to contribute in the electron transfer in the SCMFC. While on the 2^nd^ day, as observed in Fig. 3d, the SCMFC achieved its greatest values of current density and exhibited two significant oxidation peaks at 2.5 mA m^− 2^ and 4.8 mA m^− 2^ with corresponding positive sweep voltages of 0.206 V and 0.634 V, respectively. Whereas, there was only one oxidative peak at 3.14 mA m^− 2^ and 3.10 mA m^− 2^ with consistent positive voltages of 0.386 V and 0.453 V for the 1^st^ and 5^th^ d, respectively, as observed in Fig. 3c and e.

### Electrochemical impedance

Figure 3f presented the Nyquist plots and electrochemical impedance **(**EIS**)** fitting of each process, occurring at the anodic, cathodic surfaces and the whole cell. The EIS was assessed under steady state OCV conditions for SCMFC inoculated with 2% raw UMO at 41.5 g COD L^− 1^, using the anode or the cathode as the working electrode. Table S1 depicted an inset picture, representing the equivalent circuit model used to fit the anode, cathode and whole MFC impedance (two electrode’ EIS) during the stable phase, respectively. For anode EIS, it was noticed that the electron transfer resistance (R_ct_) and ohmic resistance (R_ohm_, the represented solution resistance) were 38.45 ± 3.6 Ω and 8.63 ± 0.21 Ω, respectively. Whereas for the cathode, the R_ct_ value was about 317.24 ± 12.8 Ω. Moreover, it was confirmed that the R_ct_ value of about 23.24 ± 0.27 Ω decreased in the whole MFC. Further, the entire R_int_ (including R_ct_ and R_ohm_) for bioanode was about 47.08 ± 0.22, which was lower than the cathodic R_int_ by 6.8 and about 7.87 in the whole MFC.

### Removal of UMO and bioemulsifier activity in SCMFC

Figure 3h; Table 3 display the emulsifying activity and UMO elimination in the SCMFC optimized medium supplemented with 2% UMO. The EI_24_ was raised during the operation of SCMFC over three cycles of open circuit voltage (OCV1, CCV2 and OCV3) as a result of the exponential growth of the biofilm on the anode surface. The development of this biofilm probably improves microbial absorption and bioemulsifier synthesis, which either improves the solubilization and dispersion of the oil source in the aqueous environment or makes the microbial cell surface more hydrophobic. Additionally, the CCV1 had approximately 67.17 ± 1.04% of EI_24_ activity in UMO in close circuit voltage (CCV); following this cycle, EI_24_ activity dropped to a percentage of ≥ 50% in CCV3. On the other hand, the vegetable oil’s EI_24_ rose from CCV1 to CCV3. Increased EI_24_ is correlated with UMO removal, suggesting that microbial biofilm growth on the anode promotes the production of bioemulsifiers and facilitates the degradation of UMO. This relationship is linked to both biological processes of biofilm-driven bioemulsifiers production and electrochemical mechanisms that sustain microbial activity in the SCMFC system.

### Correlation relationship between UMO concentration and the voltage output of the SCMFC

One benefit of using a mixed culture of microbes was that they synergistically degraded the UMO. Additionally, it might accelerate the rate at which substrates degrade. Remarkably, the mixed culture of microorganisms also enabled the identification of the simplest and shortest thermodynamic pathway or process, which led to a quicker oxidation of substrates. Additionally, the microorganism biofilm most likely formed, which is what caused the reduced voltage and current. According to correlation analysis, a substantial correlation was found between the various OCV and CCV parameters at a significance level of α < 0.05. The association between UMO removal and the SCMFC’s output voltage was investigated using regression analysis. The observed relationship seems to be insignificant for all parameters. Because microbial processes and other factors influence both degradation and power generation, causing biological and bioelectrochemical activity cannot accurately predict UMO removal in a SCMFC.

Referring to the OCV results presented in Table 4, the UMO removal increased as the EI_24_% with vegetable oil, EI_24_% with UMO, and output voltage increased, yielding a strong positive correlation. While in the case of CCV, the very weak negative correlation or completely uncorrelated variables were between COD removal efficiency and EI_24_% with vegetable oil. The regression (Table 4) showed that UMO removal had a negative correlation with output voltage and coulombic efficiency. EI_24_ shows that bioemulsifier production by microorganisms varies based on oil type, with weak associations from vegetable oil degradation processes. Successful mixed culture populations may not produce electricity or eliminate COD immediately. On the other hand, UMO removal had a positive correlation with COD removal efficiency. It could be inferred that UMO removal was a good predictor for output voltage, coulomb efficiency, and COD removal efficiency in SCMFC.

**Table 4:** Pearson correlation of different parameters in SCMFC

Correlations	UMO Removal (%)	EI_24_% with		Volt (mV)	COD removal efficiency (%)	Coulomb efficiency
		Vegetable oil	UMO			
OCV						
UMO Removal (%)	1.00					
EI_24_% with vegetable oil	0.99	1.00				
EI_24_% with UMO	0.95	0.91	1.00			
Volt (mV)	0.97	0.94	0.99	1.00		
CCV						
UMO Removal (%)	1.00					
EI_24_% with vegetable oil	0.48	1.00				
EI_24_% with UMO	0.24	−0.74	1.00			
Volt (mV)	−0.47	0.55	−0.97	1.00		
COD removal efficiency (%)	0.87	−0.02	0.69	−0.85	1.00	
Coulomb efficiency	−0.63	0.38	−0.91	0.98	−0.93	1.00

OCV; open circuit voltage

CCV; close circuit voltage

UMO; used motor oil

COD; chemical oxygen demand

EI_24_%; Emulsification index

The correlation coefficients for C_E_ with output voltage, and EI_24_% with UMO and COD removal efficiency were *r* = 0.98 and *r* = 0.69, respectively. The observed relationship between output voltage and EI_24_% with vegetable oil had a correlation coefficient of about 0.55. This showed that these two parameters had a moderate positive correlation. While a weak relationship was shown to exist between UMO removal and EI_24_% with UMO and also between EI_24_% with vegetable oil and C_E_%. Since the value of the correlation coefficient between EI_24_% with UMO & C_E_% is about − 0.91. It indicated that a strong correlation between two variables exists, as values of EI_24_% with UMO increase, values of C_E_% decrease. The same correlation exists between COD removal efficiency and C_E_%, with a correlation coefficient of about − 0.93. The very strong negative correlation can be explained by the COD removal affected the C_E_%. Significant oxidation of UMO by mixed bacterial cultures results in substrate breakdown as opposed to electron transfer. Microorganisms use organic matter (COD) for cell growth and maintenance and metabolize UMO components through biochemical pathways, which lowers C_E_. As the UMO oxidized, the COD removal increased; the correlation between these variables was positively correlated, *R* = 0.87 (*p* = 0.05).

### FTIR analysis

**Fig. 4:**
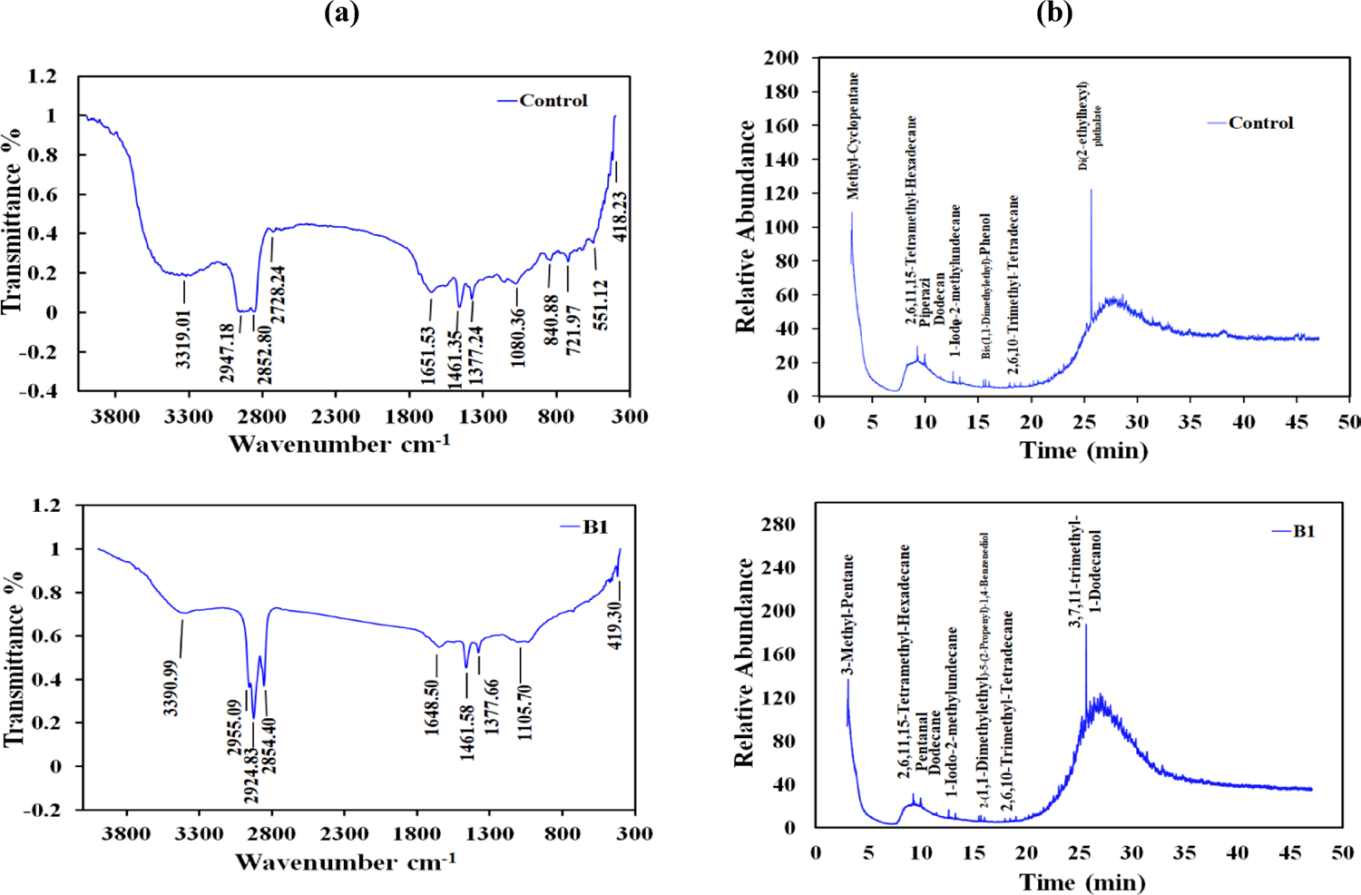

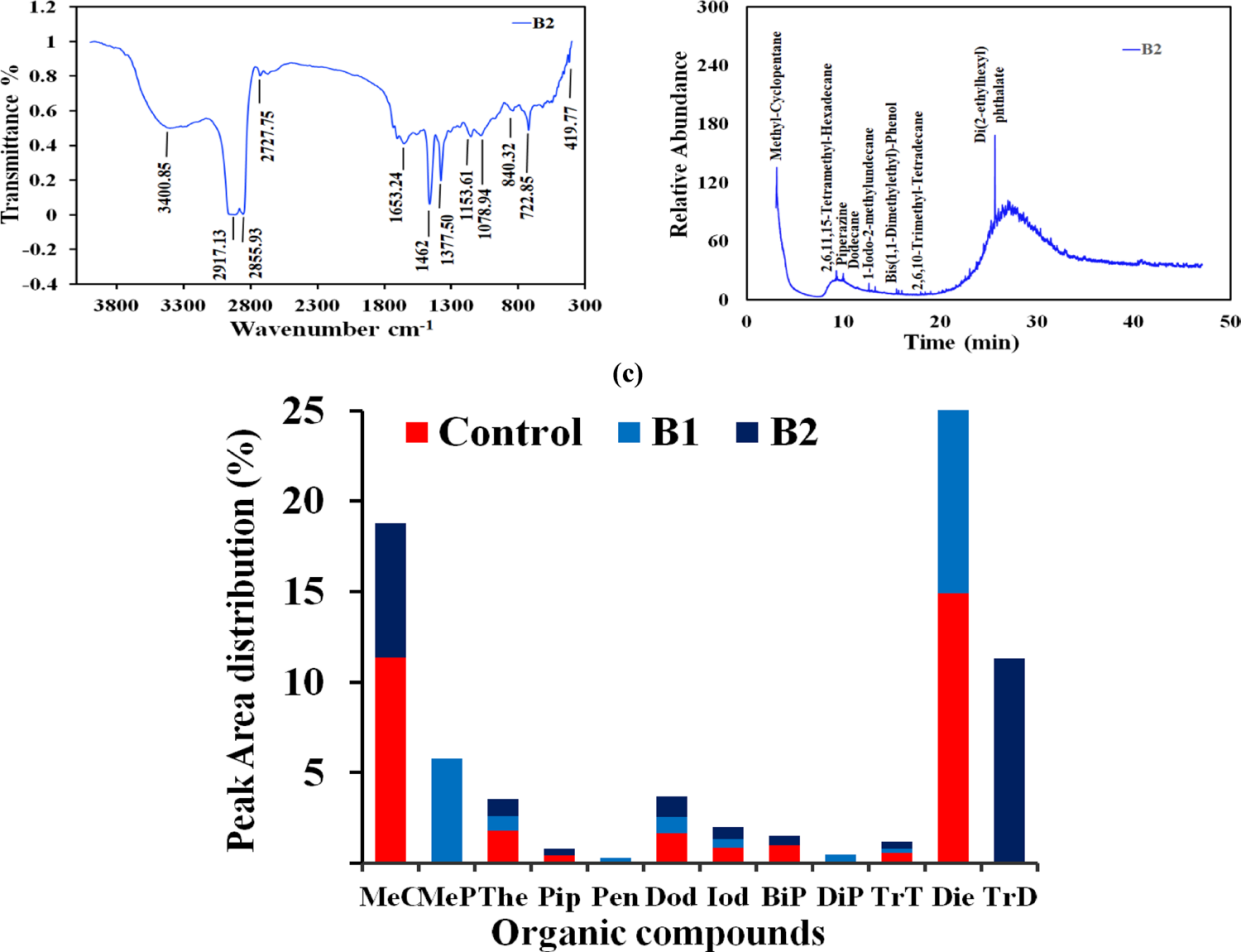
**a)** FTIR spectra, **b **GC-MS chromatogram and **c**) Distribution of organic pollutants identified on the 48^th^ (B1) and 56^th^ (B2) d of operation after the last feed replacement of the anode of SCMFC with 2% optimized UMO

Total petroleum hydrocarbons (TPHs) and polycyclic aromatic hydrocarbons (PAHs) contained in UMO were identified on the 48^th^ (B1) and 56^th^ (B2) d of operation after the last feed replacement of the anode with UMO using FTIR spectra and compared with control (before inoculation). The analysis revealed the existence of different functional groups and shows that all samples possess most of the absorption bands in the same regions with few notable differences between the bands (Fig. 4a). It can be observed that there were split bands at 2925 cm^−1^, 1460, and 1376 cm^−1^ due to the presence of a mixture of hydrocarbon compounds with small chain lengths and (C–H) branching vibrations within (–CH–) groups [19]. Moreover, the bands related to C–H vibrations of the hydrocarbon chains appear in the range 2957–2850 cm^−1^ [19, 20]. In addition, the C–H stretching bands for the control sample at 2947.18, 2852.80, and 721.97 cm^−1^, B1 sample at 2955.09, 2924.83, and 2854.40 cm^−1^ and the B2 sample at 2917.13, 2855.93, and 722.85 cm^−1^. CH_2_ scissoring and CH_3_ symmetric bending bands were observed at 1460 and 1377 cm^− 1^. While. the bands occurring in the range of 3300–3400 cm ^−1^ are related to the O–H stretching band of water or alcohols and acids that were produced from mineralization of the aliphatic and aromatic compounds of the oil during the microbial degradation process [21]. The C–O stretching vibrations for control, B1, and B2 were 1080.36 cm^−1^, 1105.70 cm^−1^, and 1153.61 & 1078.94 cm^−1^, respectively. The spectral band at 840 cm^−1^ characterized C–H in aromatic compounds. H–C=O group appears at 2728.24, 2854.40, and 2727.75 cm^−1^. Carbonyl group (C=O) stretching vibration band was observed at 1651.53 cm^−1^, 1648.50 cm^−1^, and 1653.24 cm^−1^ for control, B1, and B2 samples, respectively [19, 22–24]. The bands at 551.12 & 418.23, 419.30, and 419.77 cm^− 1^ in the control, B1, and B2 samples, respectively, were assigned to C–I [25]. The FTIR spectrum was further confirmed by GC-MS analysis.

### GC–MS analysis

GC-MS analysis of UMO samples was performed at the beginning (control), B1 (48^th^) and B2(56^th^) and revealed the presence of various chemical compounds of several TPHs and PAHs. Also, it showed the disappearance and reduction of several peaks, as well as the generation of new peaks, compared to the control. The quantification results of GC-MS analysis and the peak area distribution (%) were illustrated in Fig. 4b and c and Table S2. It could be observed that the peaks of the pretreatment (control) had a peak area% distribution higher than that of the B1 and B2 samples under the catalytic action of the microorganism in SCMFC. In addition, the UMO consisted mainly of a set of 12 peaks (C_4_ to C_24_), with intermediate branched chain hydrocarbons, cyclic and aromatic petroleum-based compounds (Fig. S1). As shown in Fig. 4c; Table 5, Methyl-cyclopentane, 2,6,11,15-tetramethyl-hexadecane, piperazine, Dodecane, 1-iodo-2-methylundecane, bis(1,1-dimethylethyl)-phenol, 2,6,10-trimethyl-tetradecane and di(2-ethylhexyl) phthalate appear to have been degraded under SCMFC operating conditions when compared with the abiotic control. Moreover, methyl-cyclopentane, piperazine, bis(1,1-dimethylethyl)-phenol and di(2-ethylhexyl) phthalate were completely decomposed during UMO biodegradation and therefore not detected in the sample of SCMFC anolyte. The Pentanal, 2-(1,1-Dimethylethyl)−5-(2-Propenyl)−1,4-Benzenediol, and 3,7,11-trimethyl-1-Dodecanol were newly detected in used biodegraded motor oil, while they were not detected in used control motor oil (before biodegradation).

**Table 5:** Quantification results of TPHs and PAHs at the 48^th^ (B1) and 56^th^ (B2) d after the last feed of SCMFC with 2% UMO and compared with before inoculation (control)

Abbreviation	Compound name	Retention time (min)	Molecular weight	Molecular formula
MeC	Methyl-Cyclopentane	3.08	84	C_6_H_12_
MeP	3-Methyl-Pentane	3.09	86	C_6_H_14_
The	2,6,11,15-Tetramethyl-Hexadecane	9.25	282	C_20_H_42_
Pip	Piperazine	9.33	86	C_4_H_10_N_2_
Pen	Pentanal	9.34	86	C_5_H_10_O
Dod	Dodecane	9.96	170	C_12_H_26_
Iod	1-Iodo-2-methylundecane	12.61	296	C_12_H_25_I
BiP	Bis(1,1-Dimethylethyl)-Phenol	15.67	206	C_14_H_22_O
DiP	2-(1,1-Dimethylethyl)−5-(2-Propenyl)−1,4-Benzenediol	15.68	206	C_13_H_18_O_2_
TrT	2,6,10-Trimethyl-Tetradecane	16	240	C_17_H_36_
Die	Di(2-ethylhexyl) phthalate	25.65	390	C_24_H_38_O_4_
TrD	3,7,11-trimethyl-1-Dodecanol	25.66	228	C_15_H_32_O

### SEM-EDX analysis

**Fig. 5:**
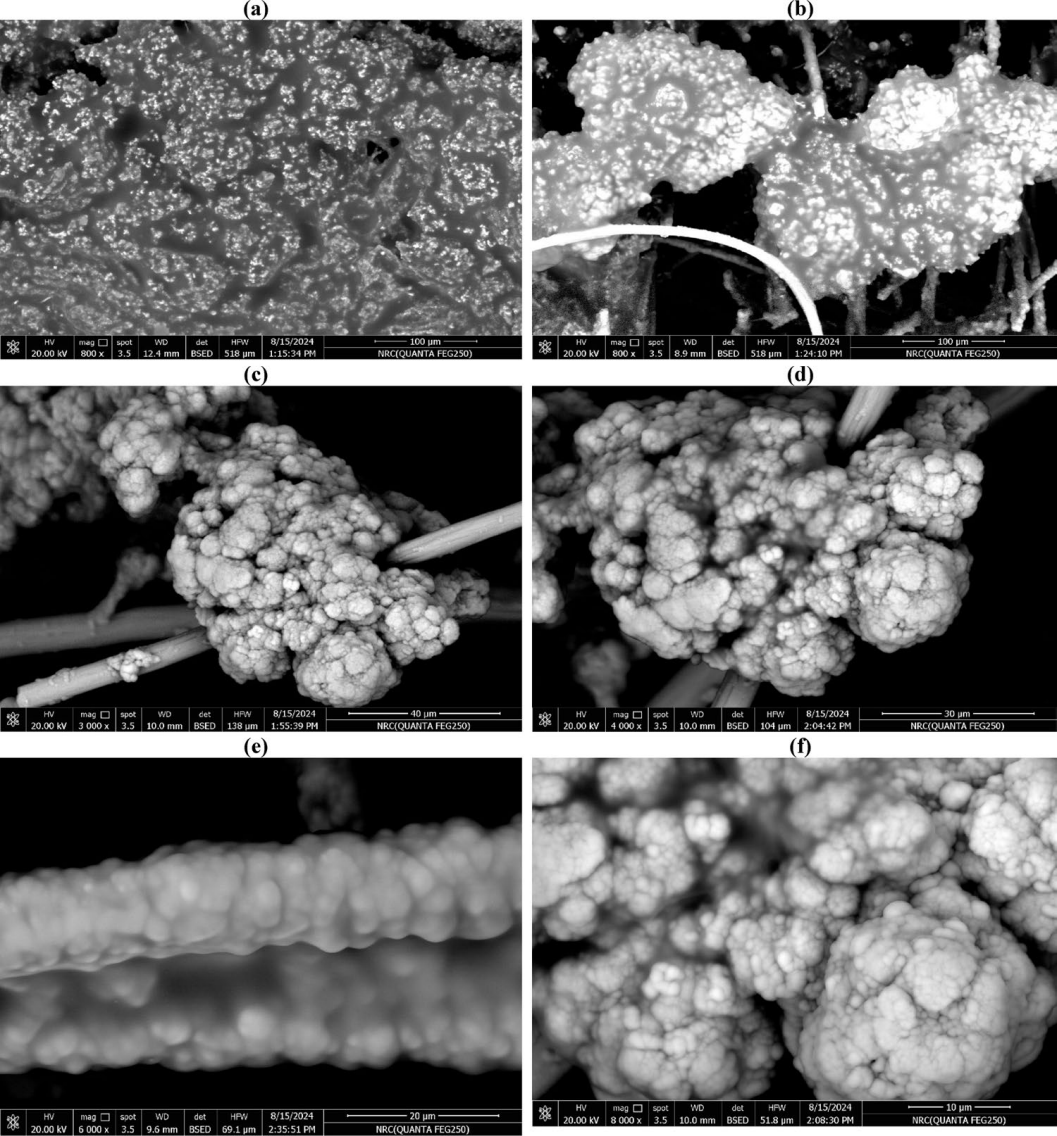
SEM analysis of anodic biofilm at different magnifications and scale bar; **a, b)** (800x, 100 μm), **c)** (3,000, 40 μm), **d**)(4,000, 30 μm), **e**)(6,000, 20 μm), and **f)** (8,000, 10 μm)

SEM image of the anodic biofilm showed a diverse group of bacteria clung to the anode’s surface, demonstrating their biocompatibility and synergy in breaking down the UMO. Furthermore, the bacterial community cells exposed to UMO appeared to have a rough surface with many white residues (Fig. 5). An EDS detector was used for additional examination, and a strong signal was found from C (Fig. S2). Members of the mixed bacterial community may have actively sequestered carbon from the MFC medium supplemented with UMO, as evidenced by the significantly higher C signal from the carbon felt biofilm.

### Taxonomic shifts in microbial communities from mixed culture to anodic biofilm during UMO degradation

To analyze the microbial community composition in each sample, amplicon sequence variants (ASVs) were obtained following DADA2-based denoising of high-quality reads from the 16 S rRNA (V3–V4 region) sequencing data. Taxonomic classification was performed using the SILVA 138.1 reference database, and relative abundance data were generated at taxonomic levels: phylum, class, order, family, genus, and species.

**Fig. 6:**
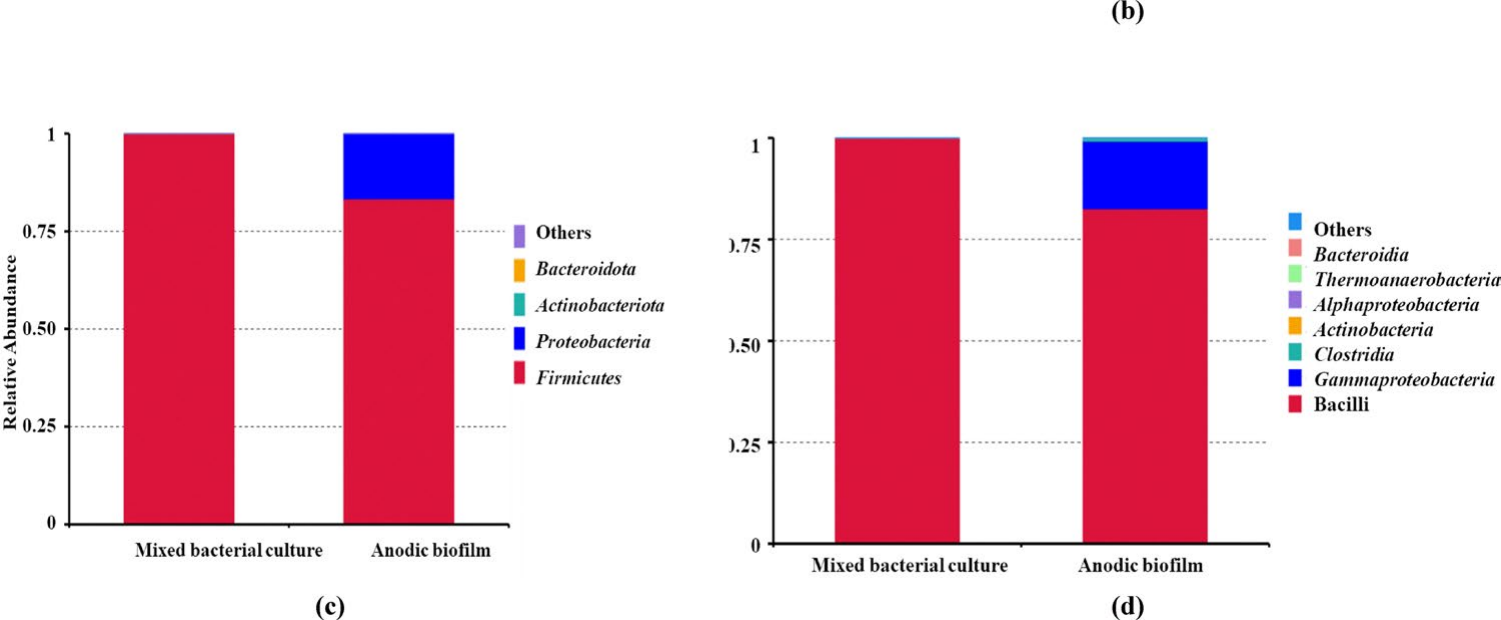

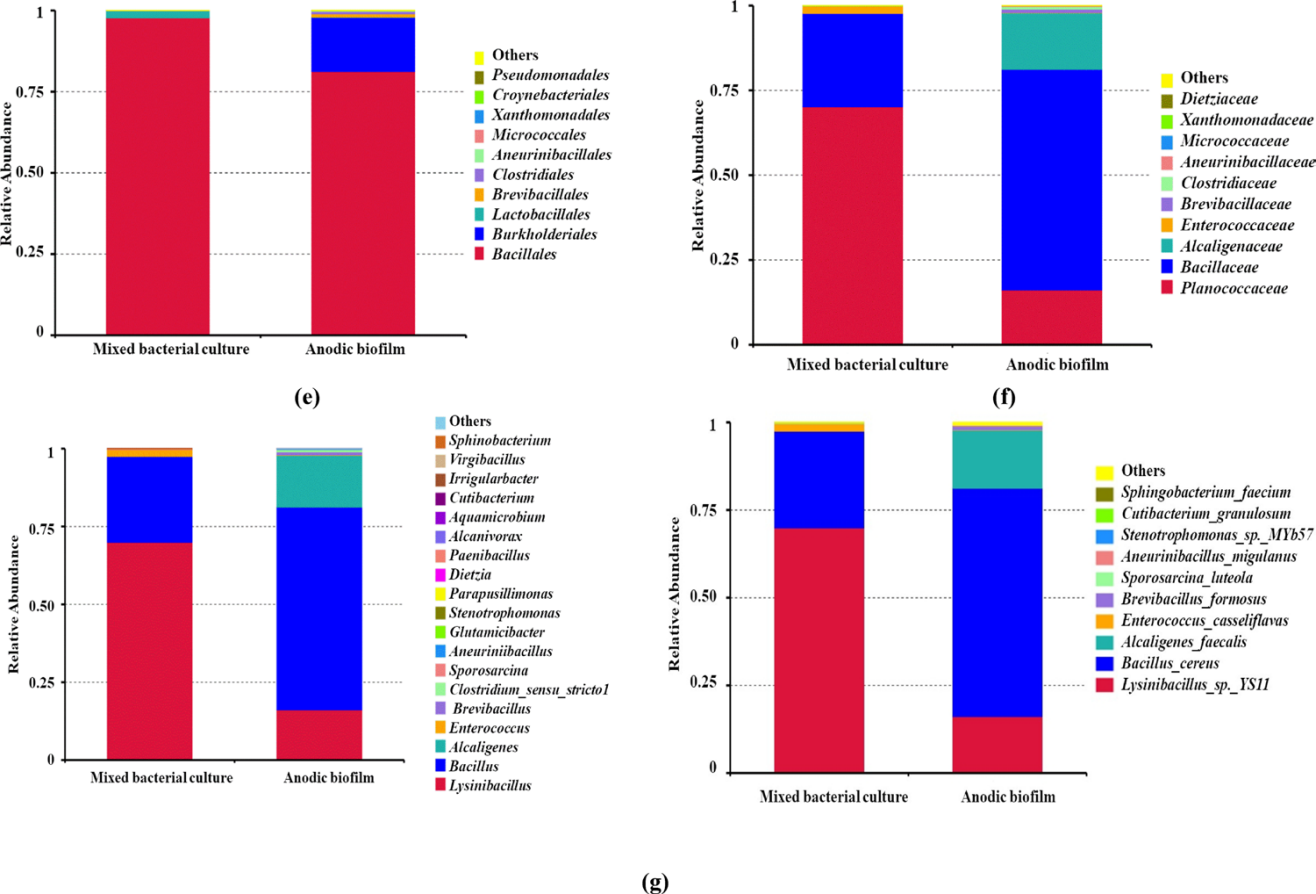

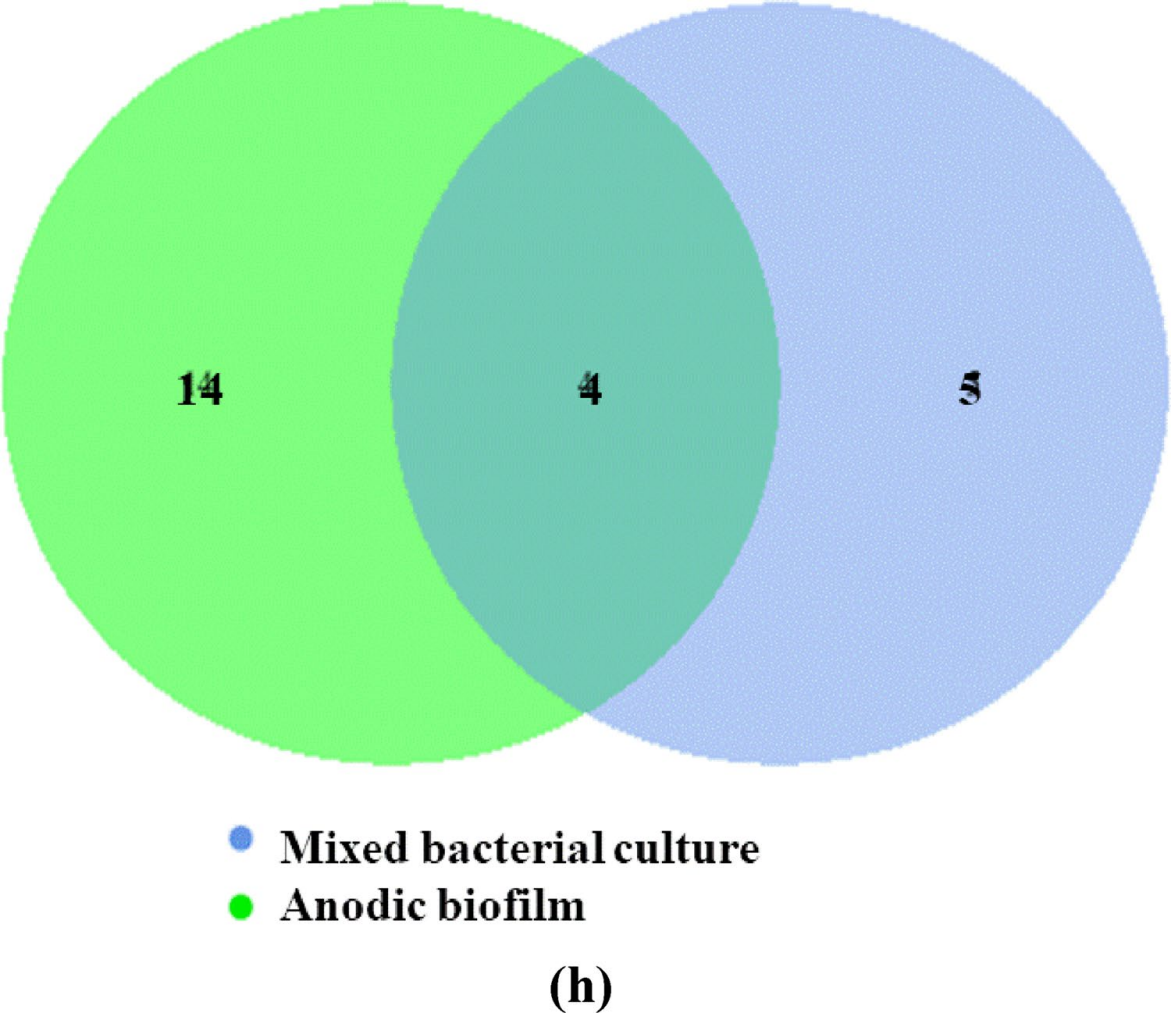

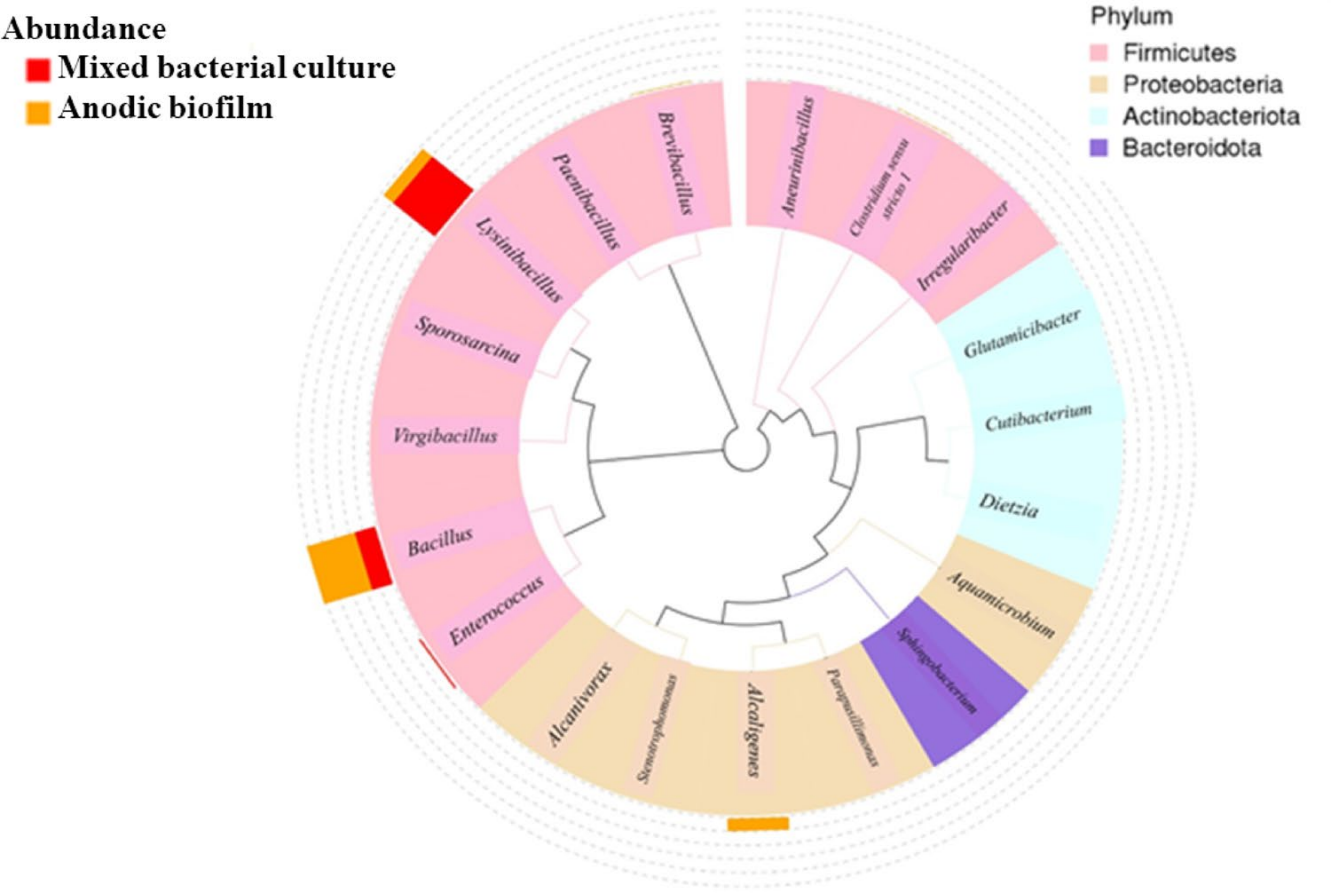
Highlights taxonomic shifts of microbial communities before and after UMO degradation in SCMFC, with notable enrichments observed in *Bacillus*, *Enterococcus*, and *Sphingobacterium* depending on the condition; **a **Taxa relative abundance in phylum level** (b) **bacterial classes** (c) **order level** (d) **family level** (e) **bacterial genera **(f)** dominant bacterial species and **(g)** Venn diagram in each circle represents one sample and** (h) **the evolutionary tree showing the taxonomic distribution and relative abundance of bacterial genera, different colors of the branches represent different phyla

Based on taxonomic annotation, the top 10 most abundant taxa at each taxonomic level were identified in the mixed bacterial culture and anodic biofilm samples. Bar charts (Fig. 6a–f) were constructed to visualize the relative abundance and shifts in microbial composition between the two conditions.

### Specific shifts in key anodic biofilm taxa

A significant taxonomic shift was observed between the initial mixed culture and the final anodic biofilm. This shift involved the reduction of the initial dominant species and the enrichment of high-performing exoelectrogenic and degradative taxa. A first key, the community transitioned away from an initial dominance by *Lysinibacillus* sp. (OTU_1, 69.94% relative abundance) toward OTU_2. Although initially classified as *Bacillus anthracis*, this sequence variant was reclassified as a non-pathogenic member of the *Bacillus cereus* group due to known limitations in 16 S rRNA gene resolution for this complex of species. This OTU became the most dominant population, increasing from 27.60% in the inoculum to a high of 65.07% in the final anodic biofilm. A second key population, OTU_3, identified as *Alcaligenes faecalis*, was undetectable in the initial inoculum (0.00% relative abundance). This species established itself successfully in the anode environment, constituting a significant 16.64% of the final biofilm community.

### Phylum-level shifts

The dramatic species-level succession was reflected in significant changes at the phylum level: The phylum Proteobacteria saw a major increase, rising from a negligible relative abundance (~ 0.03%) in the inoculum to approximately 17.04% in the final anodic biofilm. This enrichment was largely driven by the establishment of *A. faecalis* (OTU_3).

The phylum Actinobacteriota, which includes many known robust hydrocarbon degraders, also increased its presence in the anodic community, rising from a trace amount (~ 0.003%) to 1.32% in the final biofilm.

The composition of the remaining microbial community members is detailed in Fig. 6, which illustrates the relative abundance across all taxonomic levels.

The Venn diagram (Fig. 6g) illustrates the number of shared and unique feature sequences between the two samples. Four feature sequences were common, while five and fourteen unique feature sequences were specific to the mixed culture and anodic biofilm, respectively, indicating significant microbial turnover during biofilm development.

An evolutionary tree was constructed to visualize the phylogenetic distribution and relative abundance of bacterial genera in the two samples (Fig. 6h). The taxa were color-coded by phylum, including *Firmicutes*,* Proteobacteria*,* Actinobacteriota*, and *Bacteroidota*, allowing for both taxonomic resolution and ecological interpretation. The phylum *Firmicutes* dominated both environments, with prominent genera such as *Bacillus*, *Enterococcus*, *Virgibacillus*, and *Sporosarcina* prevalent in the mixed culture. These genera were known for their metabolic flexibility, spore-forming ability, and resilience in hydrocarbon-contaminated environments, which may contribute to their dominance in the liquid culture phase. In contrast, the anodic biofilm showed increased relative abundance of genera affiliated with *Proteobacteria* (e.g., *Alcaligenes*, *Aquamicrobium*), *Actinobacteriota* (e.g., *Dietzia*, *Cutibacterium*), and *Bacteroidota* (*Sphingobacterium*). These genera were often associated with biofilm formation and electroactive properties, which may favor their proliferation on anodic surfaces under electric potential conditions.

## Discussion

Microorganisms are essential to the biodegradation (bioremediation) of PHs in a contaminated environment [26]. Thus, researchers have recently discovered a notable diversity of bacteria in the soils of auto repair shops that have been contaminated by UMO [27]. Additionally, soils affected by used engine oil also had a higher concentration of oil-degrading bacteria [28]. Our bacterial mixed culture exhibited biodegradative activity consistent with other previously published findings. Mixed cultures were more effective at biodegrading the UMO because some species were able to consume the intermediate compounds generated from degradation processes and hydrocarbon wastes, while other species were able to digest all of these pollutants and their byproducts [29, 30]. An extra emulsification was often present throughout the biodegradation process of hydrocarbons, which increased the oil–water interface and accelerated the rate at which microorganisms degraded the oil [31].

Ibrahim [32] found that the ideal substrate concentration was 1% (v/v), but the results of this study did not support that finding. Abioye et al. [33] found that the largest amount utilized for the biodegradation process was between 3 and 6%. The toxicity of the UMO at concentrations greater than optimal (2%), which may have an adverse impact on the biodegradation activities of the bacterial biomass, was the cause of the mixed bacterial culture’s delayed biodegradation activity [33, 34]. The oil substrate clumped in the broth, limiting surface area and dispersion, hindering bacterial community cell attachment to hydrophobic substrates, and causing non-Newtonian behavior [35]. Used oils typically include trace components, at certain concentrations, can be harmful to a variety of organisms [36]. The metabolic capabilities and tolerances of microbial mixed cultures impact bioavailability, toxicity, and biodegradability. The ideal UMO concentration balances bioavailability and toxicity, varying based on microbial community composition and substrate characteristics.

Nitrogen was essential for the breakdown of bacterial hydrocarbons because the bacteria use it to synthesize cellular components and boost biomass [37]. At 1% concentration in our study, peptone likely provides an optimal balance of nutrients, enhancing biomass and metabolite synthesis. However, increasing peptone beyond this optimum can increase the osmotic pressure, making the medium somewhat hypertonic for cell development [38]. Additionally, certain microbes alter their cell surface to make them more receptive to hydrophobic substrates, which makes it easier for the substrates to be absorbed [39]. Considerable emulsifying activity even at 0% sodium acetate indicates the microbial capability to produce bioemulsifiers with minimal carbon supplementation when sufficient nitrogen was present, suggesting that nitrogen supply strongly influences enzyme and biosurfactant synthesis, enhancing emulsification activity.

A smaller cell population can result in fewer enzymes being secreted, whereas a bigger population causes the growth media’s oxygen and nutrients to be lost [40]. The findings of this investigation were not consistent with those of previous studies [32, 41], which revealed that the ideal inoculum sizes were 3% and 2%, respectively. Variations in the region where the bacteria were isolated and variations in their genetic composition could be the cause of the discrepancies in the results. Nevertheless, the biodegradation activity of the mixed culture was reduced above the ideal inoculum size.

The mixed culture was unable to carry out its activity due to the UMO breakdown below the incubation period of 21 d. This occurred because there were insufficient cells to break down the substrate. The findings of this investigation demonstrated that as the incubation duration increased, both UMO removal and EI_24_ levels decreased. These bacterial cultures used the hydrocarbons as a source of energy and carbon during the biodegradation process [42]. Panda et al. [43] discovered that after 5, 10, and 15 d of incubation, the biodegradation rates of 0.5% (v/v) diesel oil in water rose from 8.22% to 29.94% and 30.95%, respectively.

UMO was shown to emulsify in the culture broth, which was consistent with [44] and suggests that this mixed culture may use extracellular biosurfactant synthesis as one of its methods for utilizing UMO. The bacterial population would release surface-tension-active chemicals, while the biosurfactant producers would release compounds that aid other microorganisms in breaking down the diverse range of hydrocarbons present at the site [45, 46]. The findings suggested that the CFSs may be applied to oil recovery and hydrocarbon removal since they could efficiently emulsify both aromatic and aliphatic hydrocarbons [47]. Emulsification would make it easier to manage and transport aged crude oil [48].

The difference in UMO removal between different parameters indicated that UMO degradative activity was significantly affected by UMO conc, peptone and etc. UMO may result in modifications to degradative activity. First, UMO influenced the development and multiplication of microorganisms in their media, which in turn influenced the synthesis and release of enzymes and/or other metabolites within microorganisms. Second, a mixed bacterial culture’s growth and metabolism were also influenced by peptone, inoculum size, and incubation duration. Third, during the microbial remediation process, petroleum pollutants may directly affect microbial enzymes, either degradative inhibiting or promoting the process. The SCMFC startup period was closely correlated to the growth and activities of the bacterial communities on the anodic surface towards UMO treatment [49]. The results confirmed the formation of a dense anodic biofilm everywhere the carbon felt that already adjusted well to the environment of SCMFC to decrease the startup time for bioelectricity generation [50]. The results obtained from the current SCMFC system were compared to literature. Guo et al., [51] studied the degradation of total petroleum hydrocarbon (TPH) using sediment microbial fuel cells (SMFCs). The study showed that the COD removal rate was 55.81% and the SMFC could accelerate the catalytic degradation of PHs significantly. In addition, Mohan [52] constructed a single-chamber air cathode MFC supplied with oily sludge, and the results showed that the COD removal rate was 41.08%. Moreover, Majumder et al. [53] showed that the COD removal efficiency of oil refinery in single-chamber MFCs reached 30%. Consequently, oil refinery wastewater could be used as a substrate for electricity generation in SCMFCs. It could be summarized that the SCMFC considerably enhanced the catalytic degradation of UMO due to the highest anodic electroactive biomass concentration that subsequently metabolized the COD to produce a better-quality effluent and simultaneously produced electricity [51, 54]. It could be concluded that the consumption of soluble and insoluble organic matter UMO wastewater was associated with power output and UMO wastewater had a great potential to benefit from the use of SCMFCs to simultaneously treat UMO and produce electricity. Also, the voltage and current density of the oxidized peak were significantly increased along the operation time, demonstrating the biofilm’s gradual growth [53]. Thus, the formation of strong oxidative peaks, especially on the second day due to the development of stable mature biofilm, cellular oxidation and the production of electron and protons. Furthermore, the resulted bacterial biofilm became more electrochemically active, which could oxidize UMO to produce active oxidized compounds at maximum oxidation rate [55].

It could be confirmed that reducing both R_ct_ and R_int_ for current SCMFC reactor facilitated the movement of electrons and protons between both electrodes and more redox reactions occurring and, hence increasing in power output. Thus, it has been concluded that fitting the impedance data at constant OCV had an effect on R_ct_ and the R_int_ and determined by the SCMFC’s working conditions [56]. Consequently, the growth of mature biofilm on the anodic surface considerably decreases the anode R_ct_, signifying their catalytic role in the transfer of electrons to the anode [57]. As described above, the MFC performance towards UMO degradation occurred due to the capability of the anodic biofilm for producing a definite extracellular redox mediator that enhanced the kinetics of direct transfer of electrons between the bacterial communities and the anode, along with cathodic reactions to reduce the whole cell resistance and enhance the effective oxidation of the applied substrate.

The biofilm’s microorganisms produce bioemulsifiers, which scatter and solubilize the UMO for improved microbial uptake, as observed by the rise in EI_24_ and UMO removal during SCMFC operation. Energy generation and biodegradation were supported by the exponential growth of the biofilm on the anode surface, which also improves microbial adherence and extracellular electron transfer. Ideal biofilm conditions and bioemulsifiers production may be the cause of the first peak in emulsifying activity at CCV1, which gradually decreases with extended operation, perhaps as a result of shifting microbial community dynamics or substrate availability. Possibly due to differing microbial metabolism or surface-active chemical production dynamics in the system, vegetable oil exhibits a distinct emulsification trend that increases gradually. Higher electron transfer was not usually the result of intense decline, as indicated by the negative association found between UMO removal and output voltage and coulombic efficiency. Intermediates may be produced by UMO degradation components, which would adversely affect correlations. Although complicated metabolism promotes biodegradation and the formation of emulsifiers, which lowers electron availability, CE assesses electron capture. This results in a biological-electrochemical interaction with a decreased coulombic efficiency.

The FTIR and GC-MS analysis were conducted for further characterization of the biodegradation of UMO through SCMFC techniques. The results were in agreement with the bibliographic data, which indicated that used motor oil is a complex mixture of low and high molecular weight aliphatic and aromatic hydrocarbons (C_4_ to C_24_). These results agree well with those of Soumeya et al. [2] and Dominguez-Rosado and Pichtel [19], showed the presence of TPHs and PAHs similar to those of our work. However, it was difficult to give a precise composition of used engine oil. Indeed, its composition varies depending on the specific additives present in the oil, the type of fuel used, the mechanical condition of the engine and the length of time the oil has been used. Therefore, further research is essential to elucidate the metabolic pathways followed for the biodegradation of such compounds in those conditions. It could be concluded that the FTIR spectrum and GC-MS analysis confirmed that the electroactive bacteria were solely responsible for the biodegradation of UMO, with intermediate branched chain hydrocarbons, cyclic and aromatic petroleum-based compounds according to the appearance of newly degraded organic compounds, while they were not detected in used control motor oil.

The bacterial community’s white residues were most likely brought on by metabolites that precipitated as a result of hydrocarbon breakdown [58]. The majority of microbial cell surfaces are made up of glycoconjugates, including lipopolysaccharides, glycolipids, and glycopeptides [59], primarily in charge of the movement of contaminants across microbial membranes, the exchange of nutrients and waste products, and tolerance to external stressors. Through de novo synthesis, these cells raise the saturation of fatty acids in response to harmful hydrophobic substrates [60, 61], to maintain the fluidity and integrity of their cellular membrane and make up for the contaminants’ toxicity [62, 63].

The 16 S rRNA (V3–V4 Region) sequence analysisof the anodic biofilm in this study confirmed the strong selective pressure exerted by the UMO substrate and the SCMFC anode (Fig. 5). While the initial mixed bacterial culture was overwhelmingly dominated by *Lysinibacillus* (OTU_1, 70% relative abundance), the anodic biofilm environment rapidly selected for species characterized by superior electrochemical activity and hydrocarbon degradation capability. This resulted in a functional community structure marked by the dramatic enrichment of the *B. cereus* group and the emergence of *Alcaligenes faecalis*.

Beyond the dominance of the *B. cereus* group (OTU_2), the dramatic community shift at the phylum level provides critical insight into the functional ecology driving the SCMFC performance. The relative abundance of Proteobacteria experienced a significant enrichment, increasing from approximately 0.03% in the initial inoculum to approximately 17.04% in the final anodic biofilm. This phylum is critical as it includes many of the most well-characterized exoelectrogenic bacteria. Specifically, the emergence of *Alcaligenes faecalis* (OTU_3, a Gammaproteobacterium, reaching 16.64% is paramount, as this species is a proven anode-respiring bacterium that enhances the efficiency of extracellular electron transfer, directly contributing to the elevated power density (36.60 mW m^− 2^) and current densities observed. Simultaneously, the Actinobacteriota phylum also showed a notable enrichment, rising from a negligible 0.003% in the inoculum to approximately 1.32% in the biofilm. While a smaller fraction, this phylum is highly specialized, encompassing many robust bacteria known for their ability to degrade complex, recalcitrant hydrocarbons found in UMO. This dual enrichment of hydrocarbon degraders (Actinobacteriota) and primary exoelectrogens (Proteobacteria) establishes a stable, syntrophic association that efficiently converts the complex UMO substrate into the simpler molecules needed to power the bioelectrochemical reaction. These microbial shifts reflect both selective enrichment and functional adaptation of the community in response to UMO as a selective pressure. Hydrocarbon-degrading environments typically exert strong selective pressure that reduces overall microbial diversity while enriching for specialized taxa capable of degrading complex organic compounds [64]. The observed shift from *Lysinibacillus* to *Bacillus anthracis* and *Alcaligenes faecalis* suggests that these genera may possess superior hydrocarbon degradation capabilities and/or greater electrochemical activity under MFC conditions.

Previous studies have identified *Bacillus* and *Alcaligenes* species as important contributors to both hydrocarbon degradation and electricity generation in MFCs. *Bacillus* spp. are known for producing biosurfactants, forming robust biofilms, and secreting extracellular enzymes involved in hydrocarbon degradation [65]. *Alcaligenes faecalis* has been reported to possess strong metabolic plasticity and the ability to reduce electron acceptors, making it a common inhabitant in electroactive biofilms [66]. The increase in Gammaproteobacteria, particularly the emergence of *A. faecalis* (Order Burkholderiales), in the anodic biofilm also aligns with literature showing that members of this order are often dominant in hydrocarbon-polluted environments and contribute to complete mineralization of petroleum hydrocarbons [67]. Similarly, the emergence of *Clostridia* and *Enterococcus* suggests the development of a syntrophic microbial community capable of cooperative degradation and electron transfer processes [68].

At the species level, the appearance of *Enterococcus casseliflavus*,* Brevibacillus formosus*, and *Aneurinibacillus migulanus* in anodic biofilm implies a functional specialization of the biofilm for both degradation and electron transfer. These species are either known for their hydrocarbon-degrading enzymes or for their role in electron shuttling within bioelectrochemical systems. The increased microbial diversity observed in the optimized system was notable because functional redundancy and resilience were often enhanced in more diverse microbial communities, especially under environmental stress or when degrading complex substrates such as UMO [65]. This supports the idea that MFC not only enable biodegradation and energy recovery but also act as ecological niches that select for electroactive and pollutant-adapted microbial communities.

Importantly, these shifts also highlight the temporal dynamics of microbial adaptation, where early colonizers (e.g., *Lysinibacillus*) were gradually replaced or complemented by more efficient degraders and electroactive species as the biofilm matures. In conclusion, the metagenomic data underline the importance of monitoring microbial community dynamics in SCMFCs, both to understand the underlying biodegradation mechanisms and to guide further optimization for field-scale bioremediation of petroleum pollutants.

The study has several limitations that should be acknowledged. First, it was conducted at a laboratory scale, which may not fully represent field conditions. Second, the optimization relied on a one-variable-at-a-time (OVAT) approach, which was less robust than statistical methods like Response Surface Methodology (RSM) or Design of Experiments (DOE). Finally, field validation was necessary to assess the practical effectiveness and scalability of the microbial fuel cell system for biodegradation and bioenergy production under real environmental settings.

## Conclusion

This work demonstrates how well indigenous mixed bacterial cultures can break down and emulsify 2% UMO in a single-chamber microbial fuel cell (SCMFC), offering a practical solution for remediating UMO-contaminated areas. The observed improvements in biodegradation were attributed to high concentration of electroactive biomass and extracellular redox mediators that facilitate electron transfer. Chemical and metagenomic analyses demonstrate successful hydrocarbon breakdown and the presence of important microbial taxa. Together with bioenergy recovery, this approach provides scalable bioremediation, which might reduce environmental pollutants and generate renewable electricity. Limitations include the use of OVAT optimization, the laboratory size, and the lack of pilot-scale validations. Future researches should focus on metagenomic functional analyses to improve microbial interactions and system efficiency for real-world environmental applications, and scale up for pilot research for developed biodegradation and exoelectrogenic performance.

## Materials and methods

### Sample collection

The petroleum hydrocarbon contaminated soil sample (a mixed bacterial culture source) and used motor oil (UMO, waste) were collected from an automobile mechanic workshop in Giza Governorate, Egypt. The soil sample was collected in a sterile container in October 2023, kept cool in an icebox and transferred to the laboratory. After being collected in sterile 500 mL screw-cap glass bottles, the UMO, which had a blackish brown color, brought to the laboratory and kept in the refrigerator until it was needed. It was tyndallized for 3 h at 80 °C in a water bath over 3 d [69].

### Enrichment of mixed bacterial culture for UMO degradation

To enrich mixed bacterial cultures, 100 mL of basal mineral salts (BMS) containing 2% UMO as the only carbon and energy source was inoculated with 1.0 g of the contaminated soil sample. KH_2_PO_4_, 1; NaNO_3_, 6; K_2_HPO_4_, 1; FeSO_4_, 0.02; MgSO_4_, 0.5, and Na_2_MoO_4_, 0.02 constitute the BMS medium (g L^− 1^) at a pH of 7.0–7.2 [70]. After that, the mixture was shaken (at 180 rpm) for 7 d at 37 °C. To develop a stable and extremely promising mixed bacterial culture, successive transfers of UMO were carried out at the same concentration (20 g L^− 1^) following a 7 d incubation period.

### Optimization of UMO biodegradation assays

By adjusting the parameters one at a time, each factor that affected UMO biodegradation by the mixed bacterial culture was maximized. At 37 °C and 180 rpm of shaking, the degradation tests were conducted in 100 mL of sterile BMS medium supplemented with UMO [71]. A pre-grown mixed bacterial culture (late exponential phase growth) was centrifuged at 6000 rpm for 10 min at 4 °C, repeatedly washed (using fresh BMS), and then re-suspended in fresh BMS to prepare the inoculum. For bioaugmentation, 2% of the cell suspension was used as the inoculum. The degradation efficiency was monitored using UMO (2, 4, 6, and 8%, v/v), peptone (1, 2, 3 and 4%), sodium acetate (0, 0.5, 1.0, 1.5 and 2.0%), incubation period (7, 14 and 21 d), and different inoculum sizes (2, 4, 6 and 8%, v/v). One by one, the other parameters were optimized using the optimum conditions for the first parameter. Comparing the UMO removal to the control experiments, the amounts of total UMO decrease in BMS were calculated. The control tests did not receive any inoculation and were conducted in identical circumstances. Following incubation, samples were extracted from each flask to measure the emulsification index (EI_24_) and the percentage of UMO degradation.

All microbial work was performed under Biosafety Level 1 (BSL-1) conditions in a secure laboratory environment. Due to the known difficulty of differentiating species within the *Bacillus cereus* group solely by 16 S rRNA sequencing, and based on laboratory culture characteristics and the non-virulent nature of the environmental isolate, the culture was considered non-hazardous.

### UMO biodegradation

To extract the remaining UMO, 18 mL of chloroform: methanol (3:1; v/v) was added to the fermentation medium at the end of the incubation time. After properly mixing the solvent and culture broth, the mixture was left to settle at room temperature for 2 h and maintained in a static. The UMO-containing solvent was centrifuged for 10 min at room temperature at 6000 rpm, while the separated broth was disposed of. After separating the precipitates from the solvent, the solvent was allowed to evaporate for 3 d at room temperature [72]. Gravimetric analysis was used to determine the percentage of UMO that the mixed bacterial culture degraded as per Eq. 3.

3 $$\:\boldsymbol{O}\boldsymbol{i}\boldsymbol{l}\:\boldsymbol{d}\boldsymbol{e}\boldsymbol{g}\boldsymbol{r}\boldsymbol{a}\boldsymbol{d}\boldsymbol{a}\boldsymbol{t}\boldsymbol{i}\boldsymbol{o}\boldsymbol{n}\:\left(\%\right)\:=\:\frac{Original\:weight-Final\:weight}{Original\:weight\:}\times\:\:100$$

### Bioemulsifier activity

According to [73], the emulsification index (EI%) of the supernatants from the UMO removal experiment by the mixed bacterial community was calculated. After adding an equal amount of UMO and vegetable oil to the aqueous phase, it was violently vortexed for 2 min. The EI_24_ was then computed as a fraction of the emulsified layer height (mm), taking into account 100% of the liquid column’s total height (mm), after the mixture had been allowed to settle for 24 h. The uninoculated BMS was utilized as a negative control, while the chemical surfactant Tween 80 was employed as a positive control.

### Bioelectrochemical characterization

#### SCMFC construction and operation

SCMFC was constructed and fabricated using plexiglass material with a net working volume of 100 mL. The anode and cathode electrodes were internally faced to each other to fit inside the MFC framework. The anode was constructed from pieces of carbon felt (Fuel Cell Store, TX, USA) with effective dimensions of 2.5 × 2.5 × 0.6 cm and a projected surface area of 18.50 cm^2^. The plain cathode was made of carbon cloth with a microporous layer (6 × 6 cm each with a projected surface area of 16.63 cm^2^). The SCMFC was inoculated with mixed bacterial cultures and amended with 100 mL of optimized UMO as an energy source at an optimum maximum tolerable concentration of 2% with 1.0 g of the contaminated soil sample. The medium constituents were KH_2_PO_4_, 1 g L^− 1^; NaNO_3_, 6 g L^− 1^; K_2_HPO_4_, 1 g L^− 1^; FeSO_4_, 0.02 g L^− 1^; MgSO_4_, 0.5 g L^− 1^, and Na_2_MoO_4_, 0.02 g L^− 1^ at a pH of 7.0 at an initial concentration of 41.5 g COD L^− 1^. The SCMFC was operated under fed-batch mode, whereby the feeding was refreshed when the output voltage decreased below 50 mV, under sterile semi-anaerobic conditions. On the other hand, the cathode was subjected to air for the reduction process. Titanium wires were used as a current collector for transferring electrons and current flow between the anode and cathode electrodes. Also, the multimeter was connected to the titanium wire of the anode and cathode electrodes of the SCMFCs to record the data. At 30 ± 2C^o^, each experiment was carried out in triplicate.

#### Electrochemical measurement and analysis

The potential of the SCMFC was monitored and recorded every 5 min, using a data acquisition system (Lab Jack U6-PRO, USA). Using a resistor box (Voltcraft R-BOX 01, China), the external resistors were changed from 10 kΩ to 50 Ω in order to generate power density and polarization graphs. Furthermore, the coulombic efficiency (CE), power density, and current density were estimated in accordance with other descriptions [74, 75]. In addition, the analysis of chemical oxygen demand (COD) followed the guidelines provided in Standard Methods for Water and Wastewater Examination (APHA, 2005). Furthermore, cyclic voltammetry (CV) was performed at a workstation (Gamry, Interface 1010E, Germany) with an electrochemical electrode placed parallel to each other. The anode served as the working electrode, and the air cathode and Ag/AgCl electrode as the auxiliary and reference electrodes. CV was measured in the potential window of – 1.0 to 1.0 V vs. Ag/AgCl (3 M KCl) as reference electrode at a scan rate of 5 mVs^− 1^. Additionally, electrochemical impedance spectroscopy (EIS) of the anode, cathodes and whole cell was used to perform the electrochemical analyses of the SCMFC at a steady state open circuit voltage via an electrochemical workstation (Gamry, Interface 1010E, Germany) with a scanning frequency range of 100–0.001 kHz and 10 points per decade.

#### Fourier transform infrared spectroscopy (FTIR) analysis

UMO samples were analyzed on the 48^th^ (B1) and 56^th^ (B2) d of operation after the last feed replacement of the anode with UMO and compared with control (before inoculation) by ATR-FTIR spectroscopy to determine the functional groups and the nature of the chemical bonds by using a Bruker Vertex 80 instrument (Germany).

#### Gas chromatography-mass spectrometry (GC–MS) analysis

Further, the chemical composition of UMO samples was determined on the 48^th^ (B1) and 56^th^ (B2) d of operation after the last feed replacement of the anode with UMO and compared with control (before inoculation) by GC–MS. It was performed on a Thermo Scientific Trace 1300 series gas chromatography coupled with an ISQ 7000 single quadrupole mass spectrometer with an electron capture detector (ECD).

### Analysis the microbial composition

#### Scanning electron microscopy analysis

The anodic biofilm obtained at the end of SCMFC operation period was examined using scanning electron microscopy (SEM) along with energy-dispersive X-ray spectroscopy (EDX) (SEM Quanta FEG 250 with field emission gun, FEI Company, Netherlands). It had been fixed for 12 h at 4 °C by soaking it in 2.5% glutaraldehyde prior to examination. A series of ethanol solutions (50, 70, 80, 90, 95, and 3 times in 100%) were then used to dehydrate the sample afterthought it had been cleaned 3 times at 4 °C using 0.1 M phosphate buffer (pH 7). After drying at ambient temperature, it was sputter-coated with gold [76].

#### Microbial profiling via 16 S rRNA (V3–V4 Region) sequencing and bioinformatic analysis

Total DNA was extracted from the mixed bacterial culture used for SCMFC inoculation and anodic biofilm formation using the MO BIO PowerSoil DNA Kit (Qiagen), following the manufacturer’s instructions. The quality of the extracted DNA was assessed by electrophoresis on a 1% agarose gel, and its concentration was measured spectrophotometrically (Nanodrop, Thermo Fisher Scientific) and fluorometrically (Qubit, Thermo Fisher Scientific). The extracted DNA was used as a template to amplify the V3–V4 variable regions of the 16 S rRNA gene, using the primer pair described by [77]. Following PCR amplification, the amplicons were purified, pooled, end-repaired, A-tailed, and ligated with Illumina sequencing adapters. The resulting libraries were sequenced on an Illumina paired-end platform, generating 250 bp paired-end raw reads. Raw sequencing reads were processed using the QIIME2 pipeline (Quantitative Insights into Microbial Ecology 2). Low-quality sequences, chimeras, and adapter/primer contamination were removed using the DADA2 plugin. Sequence analysis was further performed using Uparse software (v7.0.1001, http://drive5.com/uparse/) [78]. The top 10 most abundant taxa from each sample at various taxonomic levels (phylum, class, order, family, genus, and species) were selected to generate relative abundance distribution histograms using Perl with SVG function for visualization. Venn and flower diagrams were used to visualize the shared and unique taxa between samples. Additionally, the top 100 genera with the highest relative abundance were aligned, and a phylogenetic tree was constructed using Perl scripts with SVG output.

### Statistical analysis

The data were presented as the mean ± standard deviation. One-way ANOVA and Duncan’s multiple range test (*P* ≤ 0.05) were used to assess how treatments affected UMO degradation. The ANOVA was used to assess significant differences among various treatment groups, followed by the Duncan test to identify which specific groups or treatments differ significantly from each other. Pearson correlation analysis was used to determine the rate of UMO degradation and how it related to other factors. The SPSS program (Version 15.0) was used for all analyses.

## Supplementary Information

Supplementary Material 1.

## Data Availability

No datasets were generated or analysed during the current study.
