# Supplementary material for: Biodegradation potential of used motor oil by mixed bacterial community: optimization, emulsification activity, bioelectrochemical and metagenomics analyses using single chamber microbial fuel cell
Source: Microb Cell Fact. 2025 Dec 28;25:20. doi: 10.1186/s12934-025-02889-5 (PMC12837344; doi:10.1186/s12934-025-02889-5)
Supplement: Supplementary file 1 — Supplementary Material 1. [file 12934_2025_2889_MOESM1_ESM.docx]

**Supplementary data**

**Biodegradation potential of used motor oil by mixed bacterial community: optimization, emulsification activity, bioelectrochemical and metagenomics analyses using single chamber microbial fuel cell**

**Ebtehag A E Sakr^1*,^ Nahla M Mansour^2^_,_ Hanaa M Sabaa^3^, Kamel M El-khatib^4^, and Dena Z Khater^4^**

^1^Botany Department, Faculty of Women for Arts, Science and Education, Ain Shams University, Cairo, Egypt.

^2^Gut Microbiome and Immunology Group, Chemistry of Natural and Microbial Products Department, Institute of Pharmaceutical and Drug Industries Research, National Research Centre, Dokki, Cairo, Egypt.

^3^Processes Development Department, Egyptian Petroleum Research Institute, Nasr City, Cairo, Egypt.

^4^Chemical engineering department, Engineering research and renewable energy institute, National Research Centre.

**Table S1.**

**Calculated each R_int_ and R_ct_ (R_S_ and R_ct_ of anode and cathode EIS) to whole Rint at steady state OCV condition in SCMFC and Equivalent circuit.**

| **EIS parameter** | **R_ct_ (Ω)** | **R_int_ (Ω)** | **Equivalent circuit** |
| --- | --- | --- | --- |
| **Anode** | 38.45 ± 3.60 | 47.08 ±0.22 | 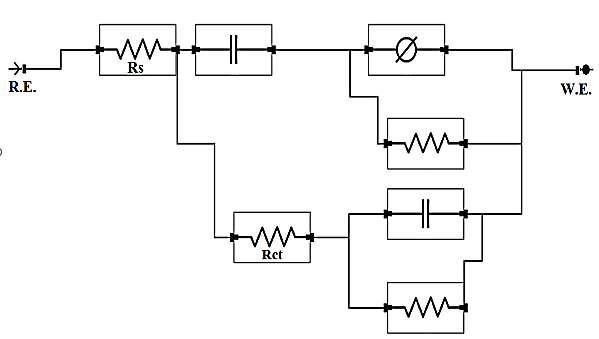 |
| **Cathode** | 317.24± 12.81 | 323.71±21.97 | 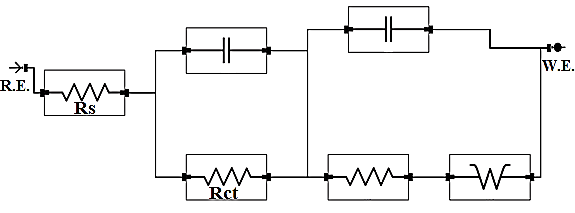 |
| **Whole SCMFC** | 23.24±0.27 | 370. 79±0.24 | 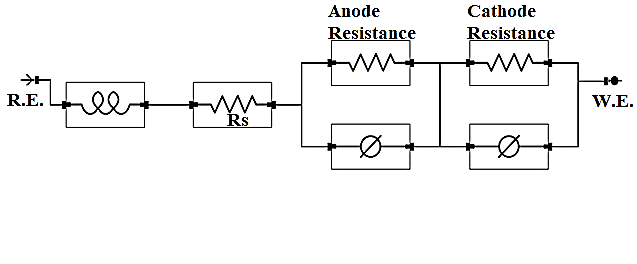 |

EIS; Electrochemical Impedance Spectroscopy

R_ct_; electron transfer resistance

R_int_; internal resistance.

SCMFC; single-chamber microbial fuel cell

**Table S2.**

**GC-MS data for the UMO biodegradation.**

| **Compound Name** | **RT** | C**ontrol**-**Area %** | **B1-Area %** | **B2-Area %** | **Molecular Weight** | **Molecular Formula** |
| --- | --- | --- | --- | --- | --- | --- |
| **Methyl-Cyclopentane** | 3.08 | 11.37 | ― | 7.42 | 84 | C_6_H_12_ |
| **3-Methyl-Pentane** | 3.09 | ― | 5.78 | ― | 86 | C_6_H_14_ |
| **2,6,11,15-Tetramethyl-Hexadecane** | 9.25 | 1.8 | 0.8 | 0.95 | 282 | C_20_H_42_ |
| **Piperazine** | 9.33 | 0.43 | ― | 0.39 | 86 | C_4_H_10_N_2_ |
| **Pentanal** | 9.34 | ― | 0.31 | ― | 86 | C_5_H_10_O |
| **Dodecane** | 9.96 | 1.68 | 0.87 | 1.15 | 170 | C_12_H_26_ |
| **1-Iodo-2-methylundecane** | 12.61 | 0.88 | 0.45 | 0.67 | 296 | C_12_H_25_I |
| **Bis(1,1-Dimethylethyl)-Phenol** | 15.67 | 0.99 | ― | 0.53 | 206 | C_14_H_22_O |
| **2-(1,1-Dimethylethyl)-5-(2-Propenyl)-1,4-Benzenediol** | 15.68 | ― | 0.5 | ― | 206 | C_13_H_18_O_2_ |
| **2,6,10-Trimethyl-Tetradecane** | 16 | 0.56 | 0.27 | 0.37 | 240 | C_17_H_36_ |
| **Di(2-ethylhexyl) phthalate** | 25.65 | 14.91 | 12 | ― | 390 | C_24_H_38_O_4_ |
| **3,7,11-trimethyl-1-Dodecanol** | 25.66 | ― | ― | 11.32 | 228 | C_15_H_32_O |

RT; Retention time

Control-Area %; before treatment

B1-Area %; after 48 days treatment of SCMFC with 2% optimized UMO

B2-Area %; after 56 days treatment of SCMFC with 2% optimized UMO


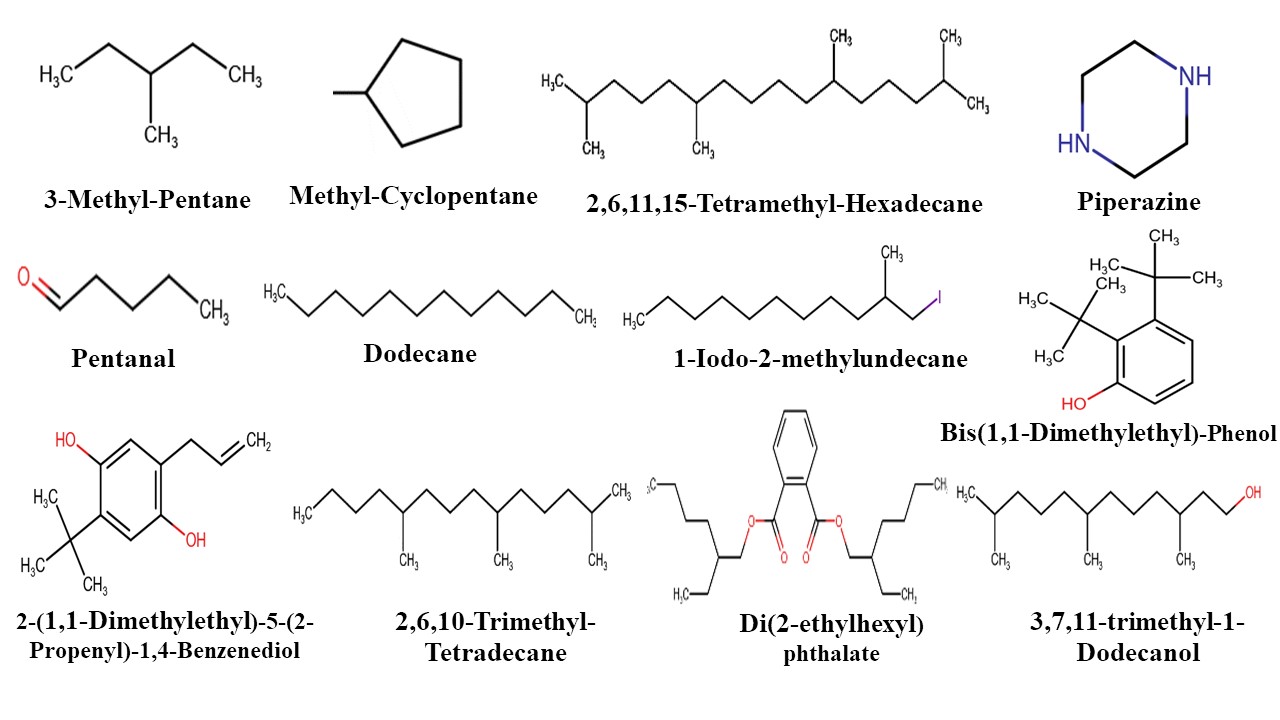


**Fig. S1. Molecular Structure of hydrocarbons detected in UMO samples; control, B1, and B2, respectively.**

| **(a)** |
| --- |
| **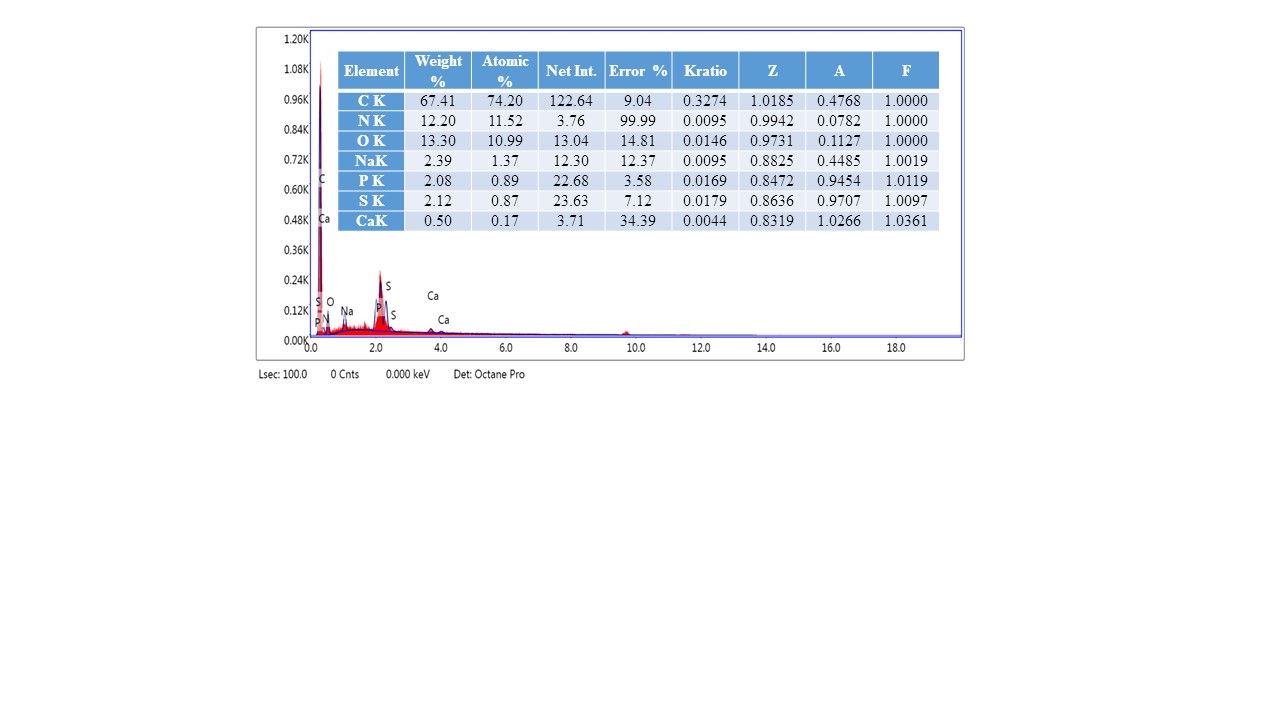** |
| **(b)** |
| **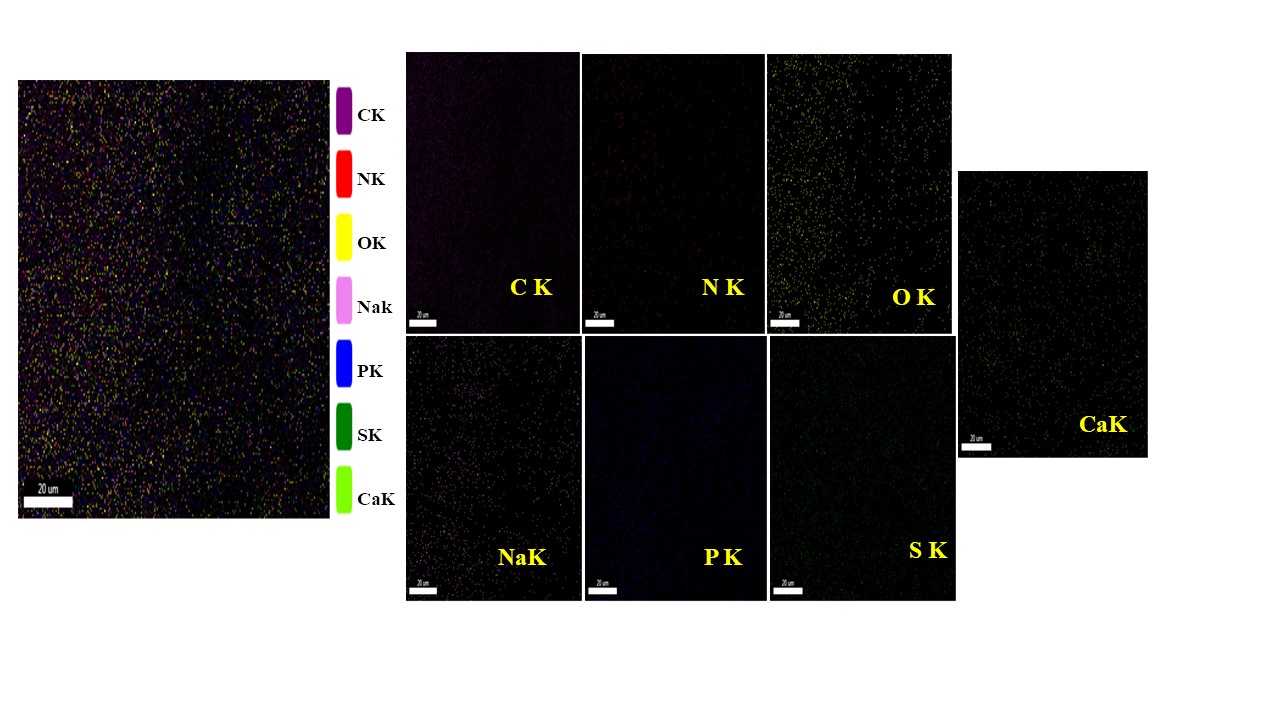** |

**Fig. S2. EDX (a), and mapping (b) of anodic biofilm.**

**Material and methods**

**Fourier Transform Infrared Spectroscopy (FTIR) Analysis**

UMO samples, before (control) and after biodegradation (B1, B2), were analyzed by ATR-FTIR spectroscopy to determine the functional groups and the nature of the chemical bonds by using a Bruker Vertex 80 instrument (Germany). A certain quantity of the UMO sample was mixed and ground with KBr in an agate mortar. The translucent discs were prepared by pressing the ground material with pressure from a bench press. The tablet was immediately analyzed with a spectrophotometer in the range of 4000-400 cm^−1^ with a resolution of 4 cm^−1^.

**Gas Chromatography-Mass Spectroscopy (GC–MS) Analysis**

The chemical composition of UMO samples was determined by GC–MS. The GC–MS analysis was performed for UMO before (control) and after biodegradation (B1, B2) on a Thermo Scientific Trace 1300 series gas chromatography coupled with an ISQ 7000 single quadrupole mass spectrometer with an electron capture detector (ECD). A 1 μL sample volume was injected by a CTC A200S autosampler with splitless mode into the DB 1 capillary separation column (60 m × 0.32 mm i.d. × 0.25 μm film thickness). For the chromatographic analysis, the initial temperature was 40 ^°^C and held for 2 min, followed by an increase at a rate of 10 ^°^C/min to the injection temperature of 290 ^°^C and was held for 20 min. The carrier gas was helium, with a constant flow rate of 1 mL/min. Mass spectrometry conditions were as follows: electron bombardment ion source, electron beam energy 70 eV, ion source temperature 210 ^°^C, transmission line temperature 280 ^°^C, mass scan range was m/z 50-650, with a scan rate was 100 ms. Data were collected on a Windows-based personal computer. The relative abundance of the components was obtained by integrating the peak area of each component in the chromatogram, and the absolute content of each component was determined by the acquisition general method.
